# Music Listening Is an Action Verb: Phoronomy, Sound Tracking and Morphodynamic Transformation

**DOI:** 10.3390/bs16071092

**Published:** 2026-07-02

**Authors:** Mark Reybrouck

**Affiliations:** 1 Musicology Research Group, Faculty of Arts, KU Leuven-University of Leuven, 3000 Leuven, Belgium; mark.reybrouck@kuleuven.be; 2 Institute for Psychoacoustics and Electronic Music (IPEM), Department of Art History, Musicology and Theatre Studies, Faculty of Arts and Philosophy, 9000 Ghent, Belgium

**Keywords:** music listening, rheology, phoronomy, sound tracking, real-time listening, morphodynamics, topological transformation, gestural approach, enaction

## Abstract

This article is a follow-up of a previous article where music was defined as matter or substance that flows. It argued for a rheological approach to music listening, conceiving of music as a virtual, motional object that evolves over time. The current article proceeds on similar lines by introducing the concept of phoronomy as the kinematic study of motion. It revolves about the way listeners can perceptually track sound by relying on motor imagery and ideomotor simulation, thus describing listening as an action verb by entailing active engagement with the sonic world. Listening, in that view, is a real-time phenomenon that keeps pace with the unfolding of the music, involving a kind of sound tracking that is characterized by sensory immediacy and perceptual bonding. By elaborating on the root metaphor of drawing an imaginary curve, it explores how listeners can stay as closely to the music as possible through a gestural approach that describes the listening experience as a virtual trajectory through virtual space. An attempt is made to provide both foundational grounding from broader theoretical frameworks and recent empirical findings.

## 1. Introduction

In a previous target article, I described music as a *fluidum* and argued for a rheological approach to music listening (Reybrouck, 2025). Music, in that view, is considered as flowing sound energy that continuously modifies its substance and shape, or in simpler terms, as movement through time. Although this may seem an obvious statement, it is not commonly translated in scholarly music studies that are usually based on static (score-based) representations of the sounds. Bridging that gap therefore is one of the major aims of this follow-up article. It is an attempt to move the rheological approach one step further by elaborating more in depth on the phoronomic approach (see below) to listening, which, then, should be defined as an action verb.

Listening, accordingly, should be considered an ongoing process of real-time interactions with the sounds. The latter can be manifest, as in the case of making music, or virtual, when mental imagery substitutes for real interactions with the sounds. There is, however, a difference in perspective when it comes to describe the music itself or the responses by the listener: where music as a stimulus can be considered an “independent variable” that can be described in an objective way, this is not the case for the interactions, which can vary greatly from listener to listener. It makes sense, therefore, to conceive of them as “dependent variables” that can be modified by numerous mediating factors. Among these are levels of processing—visceral, sensorimotor, mental or affective-emotional—as well as dispositional differences between individual listeners and contextual factors, which all can constrain or enhance the listening experience. Put in somewhat more operational terms, this means that a simple S-R model (Stimulus-Reaction model), where a stimulus elicits a reaction or response in a quasi-linear or causal way, should be expanded to an S-O-R model (stimulus-organism-response), which is a behavioral model that functions as a kind of black box model with opaque internal workings (Ashby, 1956). This model—also known as the *Mehrabian-Russell model* (Mehrabian, 1996; Mehrabian & Russell, 1974; Russell & Mehrabian, 1977)—tries to explain how external stimuli in the environment (S) may reinforce the internal emotional and cognitive state of an organism (O) and thereby influence the behavior of that organism (R). The model, even if it seems to be reductionist, has a lot of operational power by introducing the black box as a mediating factor between the objective description of the stimuli and the behavioral responses by the listener. Given that music is both a temporal and sounding art, this should mean that we can try to describe the materiality of sound (S), the attentional dynamics by the listener (O), and the resulting behavioral output (R). To let the listener stay as closely to the sounding music as possible, we may therefore propose to conceive of the music as a virtual, motional object that follows a trajectory through time while simultaneously triggering ongoing interactions with the sounds (Reybrouck, 2023).

To the extent that these interactions can be externalized as a kind of manifest or virtual motion, it is possible to conceive of them in *phoronomic* terms—see below for the term—, as a kind of tracing an imaginary curve in virtual space. Such a metaphorical projection implies a gestural reconstruction of the unfolding of the music, which could be helpful in articulating the internally felt experience by transforming the “black box” into a “glass box.” It is a phenomenological approach that entails a kind of *corporeal subjectivity* that combines both activity and passivity as the expression of a fundamental power that acts as a kind of latent tension that can be experienced in our body. This has been termed by Henry “the proper interior quivering of knowing” (Henry, 1963a, 1963b, 1965; see also Colombetti, 2014) but it also leads us to the concept of the “lived body,” as proposed originally by Husserl and adopted thereafter by Merleau-Ponty in his phenomenology of perception (Husserl, 1912/1989; Merleau-Ponty, 1945/1981). A major claim in this approach is that we have a kind of implicit awareness of our body when we orient ourselves in the world. It is a conception of the body as a “lived” rather than a merely “extended” thing, echoing the German distinction between *Körper* and *Leib*. The latter (Leib) refers to the body as experienced in the first person; the former (Körper) describes the body as described solely in physical terms. Merleau-Ponty therefore introduced the concepts of *motor intentionality* and *corporeal schema* to refer respectively to the implicit reaching out of our body (intentionality) to the world through concrete and practical activities and the implicit awareness we have of our body (schema) when orienting ourselves in the world. His insights, which initially faced considerable resistance within the scientific community, are now gaining increasing support from the field of embodied cognitive science (Pollard, 2014) by claiming that perception and thought are fundamentally structured by our bodily presence and engagement with the world. The body, in Merleau-Ponty’s view, is a subject that “acts within” rather than “observes” its environment.

In this follow-up article, I proceed along these lines by claiming that listening is an *action verb*. Listening, in that view, is not merely passive reception, but active engagement with the world. It therefore involves a processual approach that cannot be reduced to discrete acts of episodic nominalization (see below for the term) but one that presupposes a kind of “sound tracking” that is characterized by sensory immediacy and perceptual bonding. Or put in other terms: listening is first and foremost a real-time phenomenon that keeps pace with the ongoing temporal unfolding of the music.

**Figure 1:**
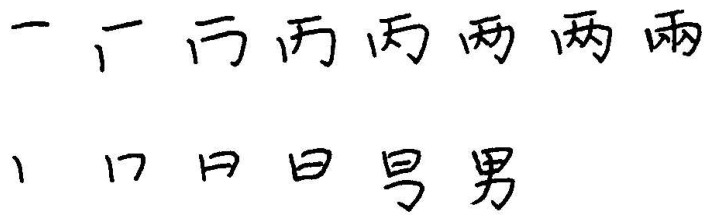
Two examples of Chinese characters, showing the successive addition of strokes from left to right to build up the final character.

To have a preliminary and intuitive understanding of this processual approach, one might find some inspiration in the art of *calligraphy*, which, as the art of beautiful writing, transforms handwriting into visual art through stylized lettering, rhythm and intentional movement, by relying on specialized tools such as brushes or pens and by using precise stroke order, pressure and balance (Andrey, 2025). Listening to music as if we are calligraphing the sounds, is an inspiring starting point. But even something more casual, like writing simple Chinese characters can be illuminating in this regard. Figure 1 provides an example. It shows how the characters are built through successive stages of adding strokes until the final character is drawn (from left to right). It is then possible to see the final character as a final and static artifact, but also in its genetic depiction as the gradual build-up of a composite figure. This build-up can be “enacted” in real time, but it can also be condensed into a simultaneous act of comprehension that compresses the successivity of the sequence into one single snapshot.

The idea of calligraphing sounds in imagination is only a starting point. What is needed first and foremost is a theoretical framework that moves from metaphorical richness to theoretical propositions that are empirically testable. To do this I start by introducing some basic foundational vocabulary that could shape future empirical frameworks in music pedagogy, sensorimotor integration, and real-time interactive design. The proposed conceptual model is quite ambitious by bridging classical metaphysics (Kantian phoronomy, Cartesian geometry, and Whiteheadian process philosophy) with modern paradigms in 4E cognitive science and structural music analysis. By reframing music listening as a dynamic, real-time kinetic gesture (“an action verb”) rather than the passive pickup of an acoustic artifact, it should provide a sorely needed alternative to static, score-bound musicology.

In an attempt to move from mere metaphoric analogies to more analytical descriptions, I first take a closer look at a number of promising conceptual analogies, such as river dynamics, density curves, solids, waterfall plots, topological transformations, and virtual multidimensional plots, to move thereafter to more operational definitions and perspectives for future empirical research. A major part of this paper therefore consists of raising questions rather than giving clear-cut answers. The questions, however, should be seen as pending hypotheses that are waiting for additional research. Here are some of them:

- Is there empirical support for conceiving of music as movement through time?
- Can we conceive of movement either as being manifest or virtual?
- Can we conceive of sound tracking as the root metaphor for active listening?
- To what extent can we compare sound tracking with the drawing of a line in virtual space?
- Is it possible to broaden the concept of a line to that of a curve or a path?
- Can we conceive of an increase in abstraction by going from a one-dimensional line over a surface in 2D-space and a volume in 3D-space to a multidimensional virtual object in an n-dimensional virtual space?
- Is it possible to visualize these imaginary curves by geometrizing the internally felt experience of being moved by the music?
- How to make the kinesthetic projection from this internally felt movement to a gestural reconstruction of the sounding music?
- How does this relate to Kant’s conception of phoronomy as a pure science of motion?
- What is the relation of phoronomy with the deictic or indexical approach?
- Is it possible to combine the rheological, phoronomic, and deictic approach in the umbrella term of listening as action verb?

The questions resume some core inquiries of the proposed theoretical framework for phoronomic listening. They are elaborated more in-depth below but provide already a first roadmap to sketch an interdisciplinary journey through the fields of Kantian phoronomy, sound tracking, morphodynamics and spectromorphological thinking, enactive cognition, gesture theory and music perception. Taken together they may be helpful in conceptualizing music listening as an active, embodied and motor imagery-based process that does justice to the dynamic character of music as a temporal and sounding art. However, this multitude of perspectives may hinder the coherence of the overall picture. I therefore provide some preliminary intuitive descriptions of some of the core theoretical constructs in Box 1, which may function as a kind of reference guide for navigating the remainder of the text. I’ve added also some possible empirical indicators, such as gesture trajectory, movement synchronization, continuous rating, physiological entrainment, or listener-report measures, to make the terms more operational. Their role in the broader model is also described shortly in the text that follows.

The theoretical constructs may seem somewhat disparate at first sight. There is, however, an overarching unity with each of them having their specific role in the proposed model. The starting point is the description of the music as something that flows over time (rheology) and that changes its shape in an ongoing and continuous way (morphodynamics, spectromorphology, topological transformations). The music, therefore, can be described as a kind of virtual object or volume (materiality of sound) that describes an imaginary displacement in virtual space (virtual trajectory). Music, however, only acquires experiential significance when it is listened to. This can happen in an active way (enactive approach) as advocated in recent cognitive models (4E cognition), with a major focus on staying as closely as possible to the sonorous unfolding (sound tracking), thus celebrating the immediacy and actuality of the sound (perceptual bonding). Taken together, this all leads to what can be considered *phoronomic listening*.

Box 1 **Overview and definitions of some of the core theoretical constructs. Possible empirical indicators (I) are listed in parentheses.**

- **Rheological listening**: conceiving of music in terms of vibrating sounding energy that flows over time (I: gesture trajectory)
- **Phoronomic listening**: tracing an imaginary line in virtual space, generalizable more broadly to the generation of a curve (I: gesture trajectory)
- **Sound tracking**: staying as closely as possible to the sonorous unfolding by keeping pace with the ongoing temporal unfolding of the music, thus showing a continuous and uninterrupted attentional focus on the sound (I: continuous rating)
- **Morphodynamic listening**: conceiving of music as a malleable substance that undergoes transformation of its shape in an ongoing and continuous way (I: gesture trajectory, listener report)
- **Spectromorphology**: the study of the temporal dynamics of the shapes, contours, and morphological characteristics of sounds, considered with respect to their spectral content (I: gesture trajectory, continuous rating)
- **Enactive approach**: the term that unifies related ideas that revolve around the concept of active sense-making by bringing forth a world of meaning through active engagement with surroundings (I: movement synchronization, physiological entrainment)
- **4E cognition**: short for embodied, embedded, extended and enacted cognition as models for the study of the way listeners cope with music as it sounds in real-time settings (I: physiological entrainment)
- **Virtual trajectory**: the mental displacement of some materiality over time that takes place at a virtual level in imagery and that highlights its trajectory as a process (I: gesture trajectory)
- **Perceptual bonding**: the real-time phenomenon of keeping pace with the unfolding music, characterized by sensory immediacy and actuality (I: continuous rating)
- **Materiality of sound**: a conception of sound as a kind of virtual object or volume that describes a trajectory in virtual space, holding the middle between a liquid and a solid without being restricted to the three dimensions of Euclidean space (I: listener report)
- **Topology**: the weakest kind of geometry, that espouses the most liberal concept of shape by dealing with the recognition of invariance on qualitative rather than quantitative grounds (I: gesture trajectory, listener report)

## 2. Music Listening as an Action Verb

*Action verbs*—also called *dynamic verbs*—describe an action that a subject or sentence performs (e.g., she *plucked* the string of a guitar) in contrast to *stative verbs*, which describe merely a state of being or perception. They can be transitive or intransitive depending on whether they require a direct object that receives the action (she plucked the *string*). Sentences with a transitive verb but without direct object, therefore, are vague and incomplete unlike intransitive verbs that do not require a direct object that receives the action (Nikolopoulou, 2023). Action verbs, further, can refer to either physical or mental actions, which means that they also imply mental representations or conceptual structures that are related to thinking, perceiving, or feeling, rather than merely being limited to behavioral events (Jackendoff, 1987, p. 153).

It is challenging to apply this to the realm of music. Especially the case of transitive verbs is thought-provoking as it is not immediately clear how music could be considered a direct object of action. Some unexpected inspiration can be found here in the scholastic writings of Thomas Aquinas who explored the concept of intentionality by distinguishing two kinds of actions, which he termed respectively “psychological” and “non-psychological” actions: some actions, such as heating and cutting, pass over to external matter (*actio transiens in obiectum*); other actions, such as understanding, perceiving, and desiring, remain within the acting person (*actio manens in agente*). Actions of the first kind bring about a state, not of the agent of the action, but of what has been changed; actions of the second kind, on the other hand, change only the state of the one who acts (Aquinas, cited in Kenny, 1984, p. 286). The distinction sounds particularly relevant today with current applications in the field of interaction dynamics between agents. Such interactions can be symmetric or asymmetric depending on whether they presuppose reciprocity between the participants of a referential exchange (symmetric) or not (asymmetric). Asymmetric interactions, therefore, are not really “inter”-actions as one of the agents acts on the other without being acted upon themselves (Reybrouck, 2023).

The concept of transitive verbs can be easily translated to music in the case of playing music, where the player’s actions are observable and manifest (e.g., bowing or plucking a string, blowing a wind instrument, hitting a key or membrane, etc.). Another obvious case is moving in response to sounding music as in dancing or simply moving along with the music in some deliberate way. Things become a little more difficult however in the case of mere listening. There is an interesting analogy here with the concepts of driver and being driven, or in more provoking terms, of *master* and *slave*. The distinction is illustrated quite clearly in the case of a basketball player who dribbles a ball. Two options are available: the dribbler lets the ball bounce free at its own frequency, or he can externally impose the frequency of bounces by forcing the ball to bounce at a rate that he determines himself. This imposed rate is the driving force that turns free bouncing into forced bouncing with the dribbler acting as a driving force. The ball then becomes a “controlled system,” while the dribbler takes the role of a “controller” who provokes a continuous disturbance to control the behavior of a coupled system (Schneck & Berger, 2010).

To translate this to the act of listening it suffices to substitute the listener for the ball. It is an obvious example of asymmetrical entrainment in the sense that the listener cannot influence the entraining rhythm of the music that acts as the driver. The music, in that case, functions as an entraining rhythm, or *Zeitgeber*—to use the German term—that triggers or forces the adjustment of an entrained rhythm to be in time with the music (Clayton et al., 2005). Things, however, aren’t as simple as they seem. There simply is no causal relation between driver and being driven in the case of music listening, given the multiple modulating and mediating factors that may intervene between the music as an eliciting stimulus and the reactions and responses by the listener. Listeners, moreover, can be open and receptive, allowing them to “resonate” in a quite natural way with the music as a vibrational art. This openness, however, should not be assumed to be the default way of listening. Listeners are free to deliberately ignore the eliciting acoustical cues or even to go against them by imposing deliberate actions on the music. The latter may be the case with listeners who can’t tap along to the music and who move independently from the music with a lack of alignment between the movement of the music and their own movements. There is, in that case, no attunement of attention, no synchronization, and no resonance (Reybrouck, 2023).

It is tempting to elaborate more in depth on the distinction between symmetric and asymmetric interactions. An interesting example is moving along with music. This can be unidirectional in the case that the listener imposes his movements on the music as a virtual agent without being receptive for the acoustic cues that come from the music itself. This reverses the role of driver and being driven, with the listener becoming the controller instead of the music. Keeping the tempo, on the contrary, even in its minute temporal adjustments, is a symmetric interaction. It implies a reciprocity of incoming and outgoing energy, as a kind of circularity between sensory input and motor output with feedback loops that continuously adjust each other in an ongoing way. It is a way of thinking that echoes the claims by the pragmatic philosopher John Dewey who stated that art unites the very same relation of doing and undergoing, of outgoing and incoming energy, that makes an experience to be an experience (Dewey, 1934/1958, p. 48). It is arguable, therefore, to advocate a way of listening that tries to externalize an internally felt experience to make it explicit both for oneself and for others. It brings us to the embodied and enactive character of active listening.

### 2.1. The Enactive and Embodied Approach to Music Listening

One way to externalize the internally felt experience of music is to take a phenomenological stance toward musical sense-making. This is exemplified most typically in the theoretical framework of *embodied* and *enactive* models for the study of the musical mind. Claiming that thinking is not solely in the brain but emerges from the interaction of the body with its environment, it describes the self-organizing aspect of cognition as an ongoing process of dynamic interactivity between an organism and its environment (Cox, 2016; Clarke, 2005; Leman, 2007; Lesaffre et al., 2017).

Such an “enactive stance,” as applied to music, is part of the emerging field of 4E cognition—short for embodied, embedded, extended and enacted cognition—as models for the study of the way listeners cope with music as it sounds (see Reybrouck, 2021 for a broad overview). It is characterized by a distinction between a mere acoustic description of the eliciting stimuli and the responses by the listener (Stepp & Turvey, 2010) with a major emphasis on the role of the sensorimotor approach to music cognition.

The enactive approach was introduced in cognitive science to unify several related ideas that revolve around the core concept of active sense-making by bringing forth a world of meaning through active engagement with the surroundings. It embraces, among others, a conception of living beings as autonomous agents, cognition as the exercise of skillful know-how in situated and embodied actions, the emergence of cognitive structures and processes from recurrent sensorimotor patterns of perception and action, a refusal to conceive of the world as a prespecified, external realm, and a view of experience as being central to any understanding of the mind (Varela et al., 1991; Thompson, 2007, p. 14). This young field of enactive cognition, however, still must come to age, as exemplified in the proliferation of conflicting and even competing theories or doctrines that use different labels to describe it (see Chemero, 2009; Colombetti, 2007, 2014; Froese et al., 2013; Gibbs, 2010; Gallagher & Zahavi, 2008; Hutto & Myin, 2013, Maiese, 2011; Noë, 2004; Shapiro, 2014; Silverman, 2013; Thompson, 2007 for an overview). This wealth of labels is a richness but is also indicative of a lack of a sufficiently comprehensive theoretical framework (see Di Paolo et al., 2010 and Reybrouck, 2020).

Central to the enactive approach is the view that giving meaning to music amounts to organizing the continuous and uninterrupted flow of experiences with the sounding world. It means that consciousness is not restricted to the brain (brain-bound) but that it also extends beyond the skin and skull. Or put in simpler terms: the mind does not stop where the external world begins, and what is outside of the body is not outside of the mind (Clark & Chalmers, 1998; Menary, 2010; Newen et al., 2018; and Schiavio & van der Schyff, 2018 for musical applications).

There is no space to go in detail here (see Reybrouck, 2021 for a broad overview). What is important for my current purposes is to bring together the concept of *enactive listening* and *dynamic interactionism* (Reybrouck, 2023) by emphasizing the role of the listener as an autonomous agent who enacts the music (s)he hears by interacting epistemically and experientially with the sounds.

### 2.2. Behavioral Analogies: Motor Intentionality and the Gestural Approach

Listening has long been regarded as a passive process of information pickup and processing that takes place mainly in the brain. This is the so-called “disembodied” approach to cognition that has been challenged so strongly by the enactive approach. Listening, however, should not merely be a kind of passive pickup but should also entail active engagement with the sounds. This is obvious in singing or playing a musical instrument with self-generated sounds that feed back to the originator of the sound-producing gestures. Each minor modification of these gestures has immediate repercussions for the produced sound, which means that the listening process is causally related to the production of the sound. It is a clear example of “extended” cognition, allowing the musician to rely on an extension of their body (the instrument) to engage with sounds either in a receptive or productive way.

The causal link between production and reception, which is obvious in this case, is less probable in the case of listening to recorded music where sound comes out of the speakers without any reference to the actual generation of the sound. The situation is a bit different in the case of a live performance, where both performers and listeners can witness the sound production in on ongoing way. Yet there is a big difference between performers who can rely on more sensory modalities—hearing the sounds and seeing and feeling the sound-producing gestures—and listeners who only can hear and see. There is a critical role here for the tactile dimension of touching the musical instrument and for the proprioceptive and kinesthetic information—kinesthesia involves the study of body motion and the perception of one’s own movement—from the musculo-skeletal system. The latter is not only related to the sound-producing actions themselves but can also refer to broader bodily feelings of tension in the muscles and the tendons, which can be shared between performers and listeners. It entails a way of listening that is related to the “embodied” approach to cognition with metaphorical projections from the kinesthetic source domain (Barsalou, 1999; Lakoff, 1993; Lakoff & Johnson, 1980).

There have been earlier attempts to capitalize on this kinesthetic sensation by bringing it in relation with inner dynamics and energetics (Kurth, 1920, 1931) and the description of music as motion (Truslit, 1938), but the biggest breakthrough came about through findings from neuroscience about the functioning of the mirror neurons that manifest similar neural activity when actions or imagined instead of being performed in a manifest way (Gallese & Goldman, 1998; Gallese et al., 2007; Molnar-Szakacs & Overy, 2006; Overy & Molnar-Szakacs, 2009; Rizzolatti & Craighero, 2004). The findings, however, are still somewhat inconclusive and controversial (see Bonini et al., 2022) but, generalizing a little, it can be stated that perception is linked to the motor system with links that work both at the neural and the behavioral level (Galantucci et al., 2006).

It makes sense, therefore, to apply the kinesthetic approach to the realm of music and to conceive of music listening in terms of *ideomotor simulation* (Prinz & Chater, 2005; Reybrouck, 2001; Shin et al., 2010), which means that perceivers can simulate the actual unfolding of a movement at a virtual level of imagery. The term has a long history in the psychological literature and has a narrow and a broader definition, but quite in general it can be stated that ideomotor theory claims a bidirectional association between the motor coding of an action and the sensory effects it produces. It also means that these associations can be used to select an action by internally activating or anticipating its perceptual consequences (Waszak et al., 2012).

My major aim in this article, however, is not merely to argue for a kind of simulation of the sound-producing gestures, but to conceive of active listening in kinesthetic terms. Kinesthesia, which has also been termed the sixth sense, allows humans to perceive movement, speed, and force of body parts by reliance on receptors in muscles, joints, and tendons. As such, it differs from proprioception which is related to static position and balance of our body.

Our body, further, is assumed to sense kinesthetically the dynamics and the temporal flow of the music (Meelberg, 2021) either as a simulation of its production—the sound-producing gestures—or as a kind of identification with the music as a virtual agent that moves through time (Reybrouck, 2025). The latter analogy, especially, has operational power. It may explain why sounds may evoke feelings of tension and resolution by literally feeling these sensations in our body, “as if” it is touched by the sounds, and by kinesthetically sensing and subsequentially processing the dynamics and physical characteristics of sound. Or as Meelberg puts it, “the materiality of sound, the manner in which sonic vibrations manifest themselves as resonances that can be felt, is kinesthetically sensed by listeners.” (Meelberg, 2021, p. 35).

It can be questioned, however, whether this is the default way of listening. There is, in fact, a distinction between musical laymen without any practice-related experience and listeners with a history of active musicianship. It can therefore be assumed that the latter engage more readily with music at a virtual level of motor or embodied simulation (Gallese, 2005). However, there are currently indications that even listeners without musical training may show activity in some motor areas of the brain. Much depends here on the way of presentation of the music, which can involve not only the auditive modality—as when listening to music that sounds through the speakers—but also the visual, the tactile and the kinesthetic one. Such multisensorial stimulation is likely to be more effective in triggering broader responses as evidenced by studies on multisensory integration, which have found an increase in neuronal response to stimuli that consists of a combination of sensory modalities as compared to responses to each modality in isolation (Pantev et al., 2009; Stein et al., 2014; Russo, 2019). It can be argued, moreover, that the effect is even more substantial when the multisensorial character of the stimuli is expanded with motor elements as in sensorimotor integration and coordination (O’Regan & Noë, 2001). It therefore seems evident that playing a musical instrument oneself can be helpful in triggering ideomotor simulation, though this is not a necessary condition for stimulating potential motor areas in the brain. Much depends here on the openness of the listener and the willingness to “resonate” almost physically with the sounds, but the individual learning history of each listener is also of paramount importance here. It underscores the importance of previous interactions with the sounds and the resulting activation of neural circuits which, in case of long-term interactions, may even lead to plastic changes in the brain (Reybrouck, 2020).

Listening to music, therefore, relies upon multiple *listening strategies*, which are partly the result of innate biological predispositions and learned and acquired reactions that reflect the listeners’ learning curves. There is, as such, a dynamic tension between phylogenetic disposition and ontogenetic development—i.e., the nature/nurture distinction. There are, in fact, motor reactions, such as the startle reflex after sudden and loud sounds, and reactive behavior in general, which occur quasi automatically, but more interesting for our current exploration are those motor responses that are controlled more deliberately. It makes sense in this regard to find out how the senses of touch and vibration are linked to motor function—as part of sensorimotor integration—and to which extent both the listener and the music can act as agents of the motor action. The latter may seem to be an arbitrary statement at first glance, but it is possible to hypostatize the music and to conceive of it as if it were a concrete, real, and independent material entity. This is an act of reification, which makes it possible to treat music as a kind of materiality that moves through time (Reybrouck, 2025) and to describe it also in physical terms. When it comes to the listener, however, things are slightly more complicated due to the tension between “manifest” movements or muscle tensions—which may indeed be small and even occur below the threshold of consciousness—and the “internalized” movements that take place in the realm of the imagination (ideomotor activity, cf. supra). Both, however, are indebted to time, so that movement can make an important contribution to the operationalization of the experience of time in general. Or as the influential American composer and writer Roger Sessions put it: “we gain our experience, our sensation of time, through movement, and it is movement, primarily, which gives it content for us” (Sessions, 1971, p. 15). The basic element of music, in this view, is not so much sound as movement: “Music is of particular significance to us as human beings because it embodies a specifically human form of movement that goes back to the roots of our being and takes shape in the inner gestures that embody our deepest and most intimate reactions.” (Sessions, 1971, p. 19). Similar ideas can be found in the writings by Sievers and Truslit who claimed that these movements and their nuances are associated with a physical act that can be projected into a physical accompanying curve (Sievers, 1924; Truslit, 1938).

## 3. Sound as Movement in Time: Morphodynamics and the Concept of Transformation

In a previous article I defined music as matter or substance that flows and argued for a *rheological* approach to music listening (Reybrouck, 2025). Rheology, which is the science that describes and assesses the flow and deformation of matter, primarily in a fluid state, can be used as an inspiration for a definition of music as flowing sound energy that continuously modifies its substance and shape. It is an interesting starting point to adhere to the materiality of sound and to conceive of music as a virtual, motional object that follows a trajectory through time.

The music, however, is only half of the story. Equally important is the role of the listener who may try to grasp the sonorous unfolding as it unfolds over time. This can be done in two ways: either by tracking the sound as a moment-to-moment history or by trying to grasp it as a comprehensive overview, somewhat analogous to the distinction Langacker made between *sequential* and *summary scanning*. Sequential scanning allows to grasp the sonorous unfolding as the successive transformations of one configuration into another with the component states being processed in series rather than in parallel. Even if their coherent experience requires a certain continuity from state to state, these are neither coexistent nor simultaneously available. What they entail on the contrary, is a kind of “processual predication” that follows the evolution of a situation through time. Summary scanning, on the contrary, is basically additive with the processing of the elements proceeding roughly in parallel. It means that all facets of a complex scene are simultaneously available in a kind of atemporal relations, thus constituting a coherent gestalt (Langacker, 1987, pp. 191, 244).

The idea that brings the music and the listening process together is the notion of *continuity* and *change*, echoing somewhat Whitehead’s process philosophy, which held that every prehension consists of three factors: the subject that is prehending—in our case this is the listener—the datum that is prehended—in our case this is the music—and the subjective form of prehending the datum. Depending on the kind of the entities he further distinguished between *physical* and *conceptual* perceptions on the one hand, and between *potentiality* and *actuality* on the other, in the sense that actuality is characterized by presentational immediacy, where continuity is concerned with what is merely potential (Whitehead, 1929/1957, pp. 28, 77). It means also that actuality is incurably atomic with the danger of spatializing the universe and ignoring the fluency by analyzing the world in terms of static categories. It is exemplified most typically in the Cartesian system of thought with its reliance on duration and measured time at the cost of subordination of fluency. The intellectual world had to wait for Newton who brought fluency back into the world, by claiming an absolute mathematical time that flows equally without regard to something external, as described in his Theory of Fluxions, which was the original term for his version of differential calculus as the mathematical approach to determine the instantaneous rate of change of moving or varying quantities (Ramati, 2001).

Much more can be said about the concept of fluency, but a major distinction was made by Whitehead, who reinterpreted Locke’s philosophy by crediting it with the implicit discovery of two kinds of fluency, namely “internal” or “external fluency,” which he termed respectively *concrescence* and *transition*. The former is the fluency that is inherent in the constitution of a particular, actual entity to become what it is, in the sense of “many becoming one”; the latter refers to the fluency by which a particular entity perishes and becomes an element in the constitution of other new entities, characterizing the passage from one element to another and how the actual becomes merely real (Whitehead, 1929/1957, pp. 242–243).

Though both kinds of fluency are distinct, it makes sense to see them also as complementary, especially in the case of music listening. The concept of transition, however, has proven problematic. William James—one of the founding fathers of psychology—has elaborated in depth on the idea of conscious transition, which he describes as the conjunctive relation by which one experience passes into another when both belong to the same self. It means that the feeling we have when a later moment of our experience succeeds an earlier one is that the transition from the one to the other is continuous (James, 1968, p. 198).

It is important, in this regard, to clearly distinguish between the fluency of an external thing and the fluency of our inner experience. As will be argued below, it may be preferable to align both approaches as closely as possible. The image that immediately comes to mind, then, is that of an *integral experience* that is characterized by a dynamic organization that needs time to complete itself, and where every successive part flows freely, without seam and without unfilled blanks into what ensues, but without scarifying the identity of the parts. This is how Dewey—another founder of pragmatic philosophy—compares the experience to a river that flows. It gives the river a definiteness and interest to its successive portions that is greater than the homogeneous portions of a pond. Or, in other words: flow in an experience goes from something to something with one part leading to another and carrying on what went before, but with each gaining distinctness in itself (Dewey, 1934/1958, p. 55).

### 3.1. From River Dynamics to Information Dynamics

The river metaphor is quite appealing. It even evokes a teleological dimension as the water, which can be compared to the materiality of sound, describes a trajectory from its starting point (the source) to the ending point (the mouth). It makes it possible to rely on the conceptual descriptive framework of *river dynamics* as a natural, ongoing process that changes the shape, speed, and behavior of the river over time and space (Okoroigwe et al., 2025). Rivers, in fact, constantly reshape themselves through processes of erosion and deposition, thus creating features such as meandering, channels, floodplains and deltas.

The analogy with music is rather obvious. Just like a river, music is a temporal phenomenon that flows and changes its shape over time. The materiality of sound, moreover, enables the establishment of relations between sounds, the listeners, and how they make sense of the sonorous unfolding. It means that vibrations can be perceived as bodily resonances that may trigger impressions, ideas and feelings, which, in turn, may resonate in the listener as a perceiving subject. Music, then, can have *narrative potential*, not merely expressed by the music, but also experienced through the listener’s bodily responses. Studying this potential, therefore, entails a definition of listening as an act in which the whole body is involved. Or, as Meelberg puts it, listening to music can be considered a form of sonic thinking with a focus on what music can do to the listener’s body and mind (Meelberg, 2021). The claims may seem a bit provocative with listeners being reduced to their body. They are in line, however, with the above-mentioned embodied approach to music cognition. To connect them with some narrative potential, however, is a major further step.

The concept of narrative, first, has multiple meanings. It is desirable, therefore, to argue for an operational definition such as the representation of a temporal development as a sequence of logically and chronologically related events (Meelberg, 2006). Such a definition is consistent with an objective conception of narrative, which is characterized by the following key features: there is a representation of events that are temporally ordered, causally related, unified, and involving actions where agents face nonroutine obstacles to the realization of their goals (Livingstone, 2009).

What stands out in this approach is the major emphasis on the inexorability of time—time considered as an intangible force that relentlessly marches forward—or put in other terms, the *temporal signature* of the narrative approach. The concept of finality is looming here with the related questions regarding the linearity and causality of the constitutive events. This finality, however, does not necessarily presuppose a “final cause” in Aristotelian sense as the ultimate goal or end for which a thing exists or is created and that represents the why behind its existence. It makes more sense, on the contrary, to adopt a Piagetian perspective and to conceive of *anticipation* as one of the universals of cognitive processing, without necessarily presupposing a final cause in the Aristotelian sense. Anticipation, in his view, must be derived exclusively from prior information either through deductive inference, motor transfer or perceptual transposition. There is, in fact, a potential confusion between the physical or physiological relation of causality (cause A produces effect B) and the logical relationships of implication (the use of A implies consequence) and instrumentality (one must use A to achieve B (Piaget, 1967, p. 225).

This reliance on previous information to anticipate what comes next has a lot of operational power, especially in the context of statistics and information dynamics. Information dynamics, in particular, studies how information evolves and flows from one moment to the next, while being processed over time and space. As opposed to static information theory which is concerned primarily with the expected behavior of “collections” of random variables, it is concerned with the information that is provided by “specific” observations at specific moments in time and focuses on the dynamic behavior of systems with a focus on how information is created, stored, transferred and utilized (Homer et al., 2024). The use of Bayesian statistics, on the other hand, is characterized by the systematic updating of beliefs and knowledge in light of new evidence, thus providing a rigorous framework for combining existing information with current, observed data to produce an updated understanding (He et al., 2025).

**Figure 2:**
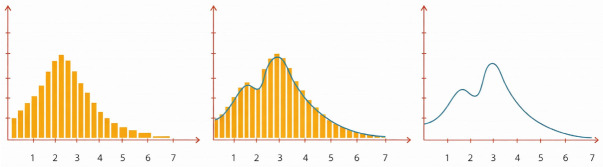
Example of a density curve (**right**) as the result of laying an envelope on the bins (**middle**) that represent the number of data points (**left**).

There is no room to go into technical details here, but combining information and frequency of occurrence is another approach with a lot of operational power. It is exemplified typically in the statistical concept of a *density curve*, which can be described in intuitive terms as the visualization of the probability of distribution of data points that can take any value in a continuum. Figure 2 depicts an example. It shows the shape of data, depicted as a smoothed graph or histogram that indicates where the values are concentrated. Depending on the numbers of bins in the histogram, the envelope that can be laid on top of the adjacent vertical bars that represent the number of data points becomes a smoothed line that gradually takes on the appearance of a *curve*.

What I present here is a rather loose description of the statistical concept of a density curve. It is only meant to serve as an inspiration for conceiving of the interchangeability of the continuity of a curve and the way it is constituted by the accretion of discrete bars. It is immediately clear that raising the numbers of the bars, and thus diminishing the width of the bins, results in a smoother histogram. It can be argued, further, that the bins should not necessarily be of equal width, and they can be filled in by any kind of content. What kind of content should be used to fill in the bars, however, is one of the major challenges of this article.

It is tempting, in this regard, to conceive of the sonorous articulation in terms of a curve in virtual space, and thinking in terms of a melodic line is clearly an obvious example. One could even go so far as to think rather naively in terms of a melody going up and down with higher notes standing for more data points than lower notes. The mere fact that singers find singing a high note more strenuous or taxing may point in the direction of an accretion of energy. One might ask, therefore, whether the melody is the ultimate bearer of form, or, put differently, to what extent it should be considered “morphophoric” in a visual representation of sounding music? Pitch and time, among other potential variable attributes of tones such as loudness, timbre and spatial location of the sound, have proven to play an essential role in this regard (Attneave, 1972; Attneave & Olson, 1971). The restriction to pitch, however, is a bit reductionist. There is, first, the question about the psychological reality of pitch as a descriptive and explanatory variable—many listeners cannot estimate the pitch of a sound—, but there is also the growing insight that pitch is a multidimensional concept, even if it is often approximated as being one-dimensional. The human perception of musical pitch, however, involves complex relationships that cannot simply be captured by one simple and linear dimension (Plack, 2005). It is worth considering, therefore, whether other attributes of the sound beyond pitch could be considered bearers of form. Alexander Truslit, who was a pioneer in music and medicine, has been quite inspiring in this regard (Truslit, 1938, see also Repp, 1993). By focusing on the relation between music and motion, he tried to provide a holistic sensation of musical expressiveness that is accessible through physiologically grounded motion patterns (Brandner, 2018). Being critical of giving a prominent role to pitch and timbre, he argued that their role is subordinate to that of *dynamics* (the subtle changes in loudness) and *agogics* (the subtle changes in tempo). What matters, then, are not the notes on a score, but the acoustic means of the dynamic development over time that function as the expression of an internally felt experience that is expressed in movement (Truslit, 1938, pp. 31, 60–63).

Truslit’s claims may seem a bit gratuitous, due to the lack of sophisticated measurement tools at that time. His insights, however, are thought-provoking (see Reybrouck, 2025 for a critical review). Especially his description of music as movement through time and its connection to the felt movement of the body, have revitalized his original ideas within the context of embodied and enactive cognition. As he put it: “The shaping of music is, in essence, the shaping of movement: thus, the sense of movement can be regarded as a sense of form.” And further: “The experience of the movement sequence can be so clear that its form can also be depicted graphically.” (Truslit, 1938, p. 45).

### 3.2. Morphology and Morphodynamics

To make these theoretical considerations more operational, let’s proceed along these lines and elaborate on two major lines: (i) the shaping of movement over time and (ii) the possibility of their depiction in some graphical way. The shaping of movement over time, moreover, can benefit from the analogy of the shaping of form in general. Two terms immediately come to mind: the concepts of *morphology* and *morphodynamics*.

Morphology, first, studies the forms of material things, either in a static or dynamic way. The latter entails interpretation in terms of force and energy while an essential part of the former lies in the comparison of related forms rather than precisely describing each of them (d’Arcy Thompson, 1917/1942, p. 19). The process of comparing, further, reduces to the recognition in one form a definite permutation of another while leaving the original figures unanalyzed or undefined. Its mathematical elaboration might find its solution in an elementary use of *the method of coordinates*, which underlies the *theory of transformations* (d’Arcy Thompson, 1917/1942, p. 1032). It is the basic method of analysis of coordinate geometry that uses numerical coordinates to map numerical pairs of tuples (e.g., x, y or x, y, z) to describe geometric shapes and to calculate distances, intersection points and slopes (see Burton, 2011; Katz, 2009; Kent & Vujakovic, 2017).

The method of coordinates was conceived by Descartes who had in mind the simple purpose of translating the form of a curve or the position of a point into numbers and words as is done every time we plot a curve. The concept of curve, moreover, has a lot of operational power as it provides a simultaneous overview of the trajectory of a point over time, showing both the trajectory of a line as a process and the lasting trace as a product. It can be considered a continuous function of time though this is not necessarily the case.

**Figure 3:**
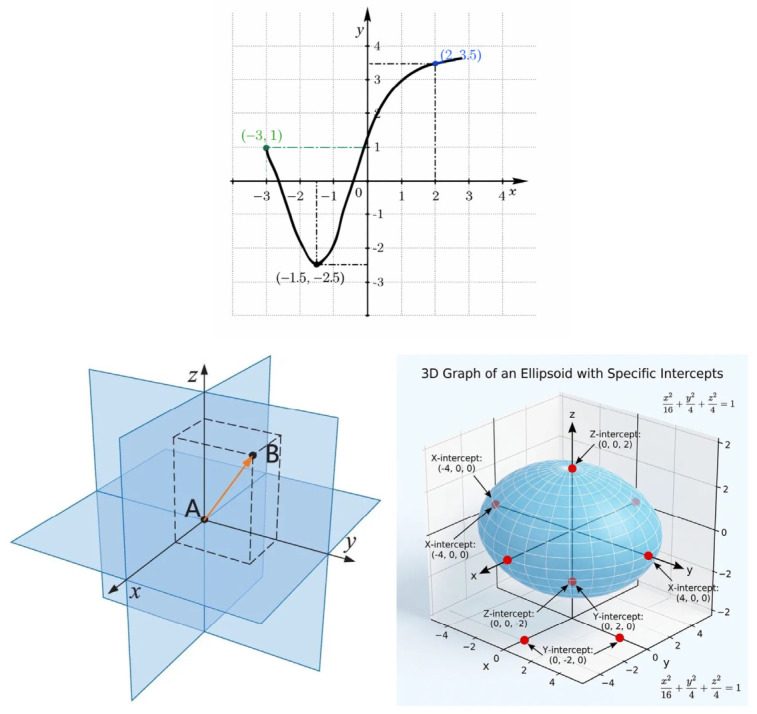
Examples of the use of coordinate systems, both in 2D and 3D representation (Reproduced and slightly adapted from Wikimedia and Easi-Peasy.AI, Copyright © Creative Commons Attribution-Share Alike 4.0 International and Easy-Peasy.AI).

**Figure 4:**
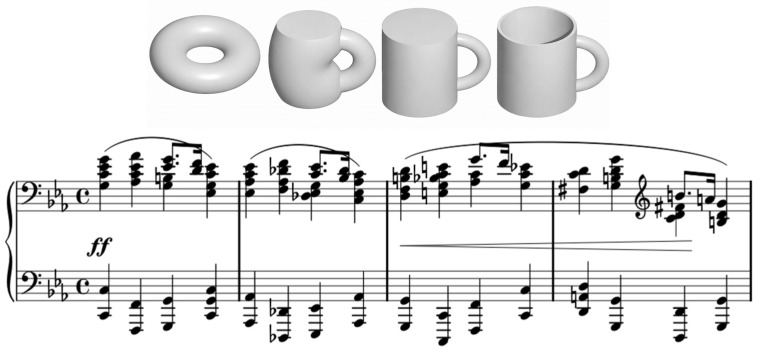
Example of a topological transformation from a doughnut/torus to a mug (**top**) (AI-generated) or from musical chords into one another, repeated four times (**bottom**). The musical example is the beginning of Chopin’s Preludes Op. 28, No 20 (Copyright © public domain).

The easiest way is to conceive of a path from a starting point to another point. This can be drawn on a two-dimensional surface (2D) with each point of the path being plotted by its x- and y-coordinates to plot a curve. Curves, however, can also be plotted in a three-dimensional coordinate system (3D), which makes the analogy with a gesture more tangible. Figure 3 provides an example. It shows the depiction of a curve in a 2D-coordinate system (top center), a cuboid in a 3D-coordinate system (bottom left), and a 3D graph of an ellipsoid with specific intercepts (bottom right). The ellipsoid, in particular, is inspiring because it can be considered a three-dimensional solid figure or a closed surface that bounds a solid. It can be filled, remain empty inside, or be partially filled. The idea of a solid, however, is quite appealing as it entails the possibility of thinking of it in plastic terms and to change its form. The example of the topological transformation of the doughnut/torus-mug (see Figure 4) homeomorphism immediately comes to mind here, but other examples could work just as well. At a higher level of abstraction, however, it is even possible to conceive of an n-dimensional virtual object that moves in virtual space (see below).

What really matters here is the transition from a one-dimensional, over a two-dimensional to a three-dimensional approach in imaging the shaping of the sound. It challenges a reductionist conception of thinking of music in terms of a melodic line in favor of a trajectory of some materiality that holds the middle between a liquid and a solid (see Reybrouck, 2025). The term voice-leading might be somewhat more promising here as it refers to the technique of arranging musical voices so that individual melodic lines move smoothly from one configuration to another typically using minimal motion. But still more interesting are chord progressions with chords that transform into each other by choosing the shortest paths between the notes. By focusing on the chords as configurations of single notes, it is possible to think in plastic terms of chords as quasi-solids that may be experienced as voluminous sonorous masses. Figure 4 provides an example of this analogy. It shows the first four bars of Chopin’s Preludes Op. 28, No 20, showing four repetitions of a 4 chord-progression that can be experienced as the plastic transformation of the chords into one another, somewhat analogous to the donut-mug transformation;

There is an interesting analogy here with an educational application in Hindustani Dhrupad singing, which is characterized by smooth, slow melodic glides, whereby pitch is conceived as a continuum. Even if the melody involves the precise and subtle intonation of discrete notes, it is also experienced as the movement through paths, trajectories, and shapes in an imaginary pitch space. It is an approach to singing that highlights the significance of the space between the notes as being even more important than the individual notes themselves (Fatone et al., 2011; Van der Meer & Rao, 2006). As Dhrupad vocalizers themselves point out, they smoothly navigate within an imaginary pitch space while simultaneously mirroring this navigation by corresponding hand movement in the real three-dimensional space, showing a systematic relationship between hand movements and melodic phrases and patterns (Clayton, 2017; Leante, 2009; Paschalidou, 2022; Rahaim, 2012). It is a connection that goes beyond mere spatial 3D Euclidean geometry by extending it to the sensation of forces in the senses that vocalists seem to engage with the music through manual interactions with imaginary objects and whereby motor imagery is “materialized” through effortful physical actions that are directed towards these objects. As Paschalidou puts it: “Singers appear to engage melody and the melodic modes by manually sculpting spaces as if they were physically tangible, posing a distinct sense of resistance. These spaces are conceived as consisting of pervasive substances, such as an elastic band, a ball, water, etc., that afford various interactions such as stretching, expansion, compression, pulling, throwing, etc.” (Paschalidou, 2024, p. 3). It is an instructive example of the special role of spatial perception and haptic sense in Dhrupad music pedagogy.

**Figure 5:**
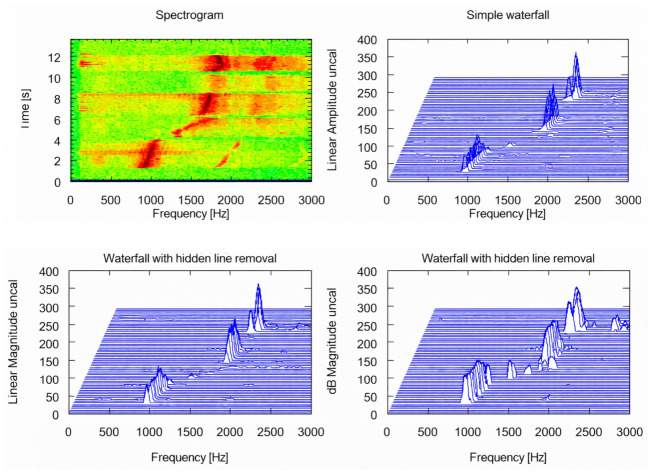
Depiction of a spectrogram (upper left) and 3 styles of waterfall plots of a whistled sequence of 3 notes vs. time (Slightly adapted from https://commons.wikimedia.org/wiki/File:Waterfall_plot_of_a_whistle.png, accessed on 22 April 2026, Copyright © GNU Free Documentation License).

To generalize from Dhrupad pedagogy to broader musical applications, it can be argued that the emphasis on pitch and melodic patterns is perhaps too limited, given the multidimensional character of pitch but also of music in general. It may seem that the concept of the materiality of sound as a kind of virtual object that describes a trajectory in virtual space has more operational power. Conceiving of virtual space, moreover, also allows to go beyond the restrictions of 3D Euclidean space, and to conceive of sound as existing in a *multidimensional space*. I therefore propose to conceive of the bearers of form in a more holistic and abstract way as the materialization of sound that is moving and changing it shape through time. I am inclined, moreover, to go even further in abstracting by also introducing the visual tools of waterfall plots of spectrograms as a representation of the imaginary objects that move through time. The spectral configuration at each moment—measured by a short temporal window as in Fast Fourier Transform—could then represent the imaginary material sounding objects that might change their shape—in this case their spectral content—of the vibrational sounding result. Figure 5 depicts the example of a whistled sequence of three notes, first depicted as a spectrogram, and then showing three waterfall plots of the same sequence of sounds (simple waterfall depiction and depiction with removal of hidden lines, either with linear amplitude and dB magnitude).

Waterfall plots are three-dimensional visualization tools that show how two-dimensional phenomena change over time. This is a key difference from solids, which generally remain stable over time. They display multiple curves of data, such as spectra, which are spread simultaneously across the screen and vertically with nearer curves masking those behind, resulting in a kind of mountain shapes that appear side by side. They are conveniently used to display spectrograms, which are the result of calculating the frequency spectrum of a compound signal such as music. In more intuitive terms they can be considered a three-dimensional plot of the energy of the frequency distribution of a signal that changes over time.

It is tempting to define these spectrographic visualizations of sound as potential bearers of form either in a discrete or continuous way. As mentioned already in my previous target article (Reybrouck, 2025), I have argued for a definition of music as a *fluidum*, conceiving of it as flowing sound energy that continuously modifies its substance and shape. Some time intervals (time windows) of the sonorous unfolding may take on a recognizable form that characterizes them as separate and identifiable units with some semantic weight, while others may function as transitions between these units. There is, as such, a dynamic tension between discrete and continuous processing of the sound signal, which also means that the spectral configuration of the sound, together with its loudness parameters, can function as the substrate for a *morphodynamic* conception of form.

**Figure 6:**
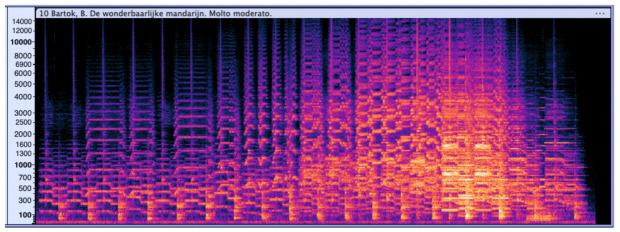
Example of a spectrographic depiction of Bela Bartók’s pantomime ballet The Miraculous Mandarin (Molto moderato) with repeated and slightly modified patterns of spectral configurations.

An interesting contribution in this regard is Cogan’s conception of *spectral morphology* of different sonic moments. As he puts it: “morphologies may resemble one another, may be transformations of one another, may oppose one another—or may do all those three at once. In these ways the different sonic morphologies interrelate, and out of these interrelationships emerges a spectral pattern, design, or shape that is an entire musical piece.” (Cogan, 1984, p. 124). Figure 6 shows an example. It depicts the 10th section (Molto moderato) of Bela Bartók’s pantomime ballet The Miraculous Mandarin. It provides a clear overview of the dynamic development of an extended temporal unfolding over time (1.36 min) with recognizable parts that are partly repeated and partly modified.

A related approach is Smalley’s concept of *spectromorphology*, which studies the dynamic shapes, contours, and morphological characteristics of sounds, considered with respect to their spectral content. It reflects the temporal dynamics of sound that reveals its evolution and structure, shaped by articulation, internal coherence, and temporal changes. The term was coined to describe the spectral content of a sound, or, in more technical terms, the occupancy of spectral space and spectral density (Smalley, 1997). It sets out spectral and morphological models and processes and provides an operational framework for understanding structural relations and behaviors that can be experienced in the temporal flux of the sounding music (Smalley (1986)). As such, it concentrates on the spectrum of available pitches and their shaping in time, which Smalley describes as a “collection of tools for describing sound shapes, structures, and relationships, and for thinking about certain semiotic aspects—potentially analysis of a kind” (Smalley, 2010, p. 95).

The spectromorphological tool can be used to describe and analyze the listening experience. Its overall morphological framework can be summarized by three morphological states within the amplitude envelope of the sound: the onsets, the functions for continuation and termination, and the ways how the sound evolves, along with an emphasis on processes of motion and growth and their variations as applied to fine-grained or larger levels of the musical structure (micro and macro levels). As Smalley puts it: “A gesture is therefore an energy–motion trajectory which excites the sounding body, creating spectromorphological life. From the viewpoint of both agent and watching listener, the musical gesture-process is tactile and visual as well as aural. Moreover, it is proprioceptive: that is, it is concerned with the tension and relaxation of muscles, with effort and resistance. In this way sound-making is linked to more comprehensive sensorimotor and psychological experience” (Smalley, 1997, p. 111).

The spectral approach to music is a promising approach. It challenges the restrictive approach to musical form in terms of melody and melodic patterns with empirical support from the fields of neuroscience and mathematics who describe music processing in terms *of spectrotemporal modulation frameworks*. It is a new approach that allows the decomposition of a complex stimulus into simpler spectral and temporal modulation functions, somewhat similar to the use of visual gratings in visual research to model complex scenes (Chi et al., 2005). It is arguable, therefore, to represent sounds in a spectrotemporal modulations space rather than representing them merely in terms of frequency as a function of time. Both kinds of representation, however, can be related to each other by the mathematical relationship of *Fourier transformation* (Shamma, 2001).

The spectrotemporal description of music thus has a lot of operational power. It allows to visualize the sound either in a 2D or 3D coordination frame, adhering to the technique of media translation that transforms the auditory domain into the visual. Much inspiration can be found here in experiments involving sonification of three-dimensional objects (Blow, 2014; Battey, 2015; Mannone, 2019) with simultaneous perception of sounds and their representations as 3D-objects. What we should have in mind, however, is the inverse process from sound to 3D-images.

The idea of 3D-objects and more in particular of moving objects that evolve over time while changing their shape, has a lot of descriptive power for the metaphoric projection of the materialization of sound. It reinforces the idea of music as a *plastic art* in the original etymological meaning of the word (from the Greek πλαστικός (plastikos = capable of being molded or shaped)). Music, in this view, could be conceived as a kind of malleable substance that undergoes transformation of its shape in an ongoing and continuous way. The concept of *morphodynamics*, as introduced by René Thom and adapted by Jean Petitot, immediately comes to mind here. By contrasting his dynamic conception with that of a mechanism, he argued that “physical objectivity” is not amorphous (Thom, 1989, p. 112). The same also holds for “internal mental physics,” which he viewed as a continuous reconfiguration of the sensory world within the dynamic process of cognition and as the emergence of structures that arise from underlying neuronal dynamics (see Petitot, 1992; Petitot, 2009; see also Di Iorio, 2024; Franceschelli, 2024).

### 3.3. Sound as Movement in Time: From Curve to Path to Gesture

The above may sound somewhat abstract. The connection to our main line of thought is however evident. The introduction of a teleological element (from the Greek τέλος (telos = end, goal, purpose, completion, or fulfillment)), in fact, entails to some extent the concept of motion and, even more specifically, of *locomotion*. The latter implies a trajectory that goes from one place to another, implying a spatial connection of distinct locations and the possibility of traveling between them. A distinction should be made, however, between the actual “physical” displacement in real time and space, and the “mental” displacement that takes place at a virtual level in imagery. Music listening, in particular, may induce a feeling of forward movement with the equally challenging question: What is it exactly that moves? Is it the music itself, or the listener who moves along with the music in a virtual world of *ideomotor simulation* (see Reybrouck, 2025 for the term)?

The questions are difficult to answer as there are many ways of listening and also many listeners who, to a greater or lesser extent, can activate their motor responses to the music. What I argue for, however, is an attempt to apply the morphodynamic framework to the concept of *sound tracking* and its *gestural analogy*. Sound tracking, as I define it, simply means that the listener stays as close to the sonorous unfolding as possible, thus showing a continuous and uninterrupted attentional focus on the sound. It is an approach that conceives of music as having the gestural force of a resembling object, both animate or not. By extension through analogy, we can then legitimately adopt the root metaphor of the sonorous object as a sound-producing organism and conceive of the gestures made by that musical organism (Coker, 1972, pp. 17–18).

A critical factor in this analogy is the simultaneous alignment of the listener’s attention with the unfolding of the music “as if” the listener is tracing a virtual curve in virtual space. It is an obvious example of sensorimotor integration with the music as an incoming sensory stimulus leading to a motor reaction that can be both internalized or manifest. Many questions are still open here, however: Should the movement mimic the movements of the music or should it be considered as the externalization of an internally felt experience by the listener? Interesting examples here are the movements of conductors and the accompanying bodily gestures by performers or listeners (see Godøy & Leman, 2010; Gritten & King, 2013; Hatten, 2004 for a broad overview). As suggested by the title of this contribution—music listening is an action verb—I would like to introduce the concept of *phoronomic listening* here, which I will define below.

**Figure 7:**
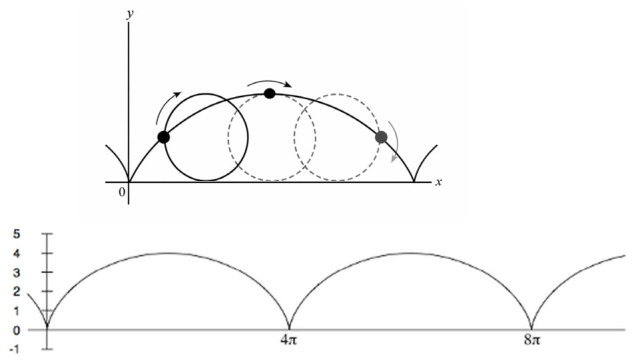
Depiction of the generation of a cycloid by the movement of a point along the circumference of a circle while rolling along a straight line (Copyright © Wikidot 2025, http://mathonline.wikidot.com/the-cycloid, accessed on 9 February 2025).

I take as a starting point the dynamic aspect in the metaphorical projection of a *line* that can be generalized more broadly to the generation of a *curve*. The latter can be regarded as being engendered by the movement of a point that is subjected to some lawful constraints. Examples are plane curves that are defined in terms such as a circle, ellipse or hyperbola, parabola, spiral, cycloid, etc. (Comte, 1830–1842/1998, p. 161). Figure 7 shows the example of a cycloid as a special kind of a curve that is traced out by a point on the circumference of the circle as it rolls along a straight line.

Two aspects are important in the generation of this cycloid: the genesis of the curve as a process that unfolds in time and the constraints that give it some kind of lawfulness. The latter especially might be important in case of converting an “internally felt” movement while listening to music into some kind of “motivated” and/or “externalized” movement. Listeners who are unable to keep up with the beat are typical examples of how unmotivated gestures can lack any causal relationships between the music and its evoked motor induction. It makes a lot of sense, therefore, to explore the possibilities of gestural translation of music as a trajectory through time.

Such a trajectory can be of any length, going from a short motif of a few seconds to larger temporal spans that can last for more than an hour, as in the case of the large symphonic works of the romantic period in European classical music. But even in the latter case, these larger spans can be subdivided in shorter spans, which can be described in metaphorical terms as *path functions* that start at a specific point in time and space and end at another point that functions as a goal. Such a metaphorical projection has been termed the *source–path–goal schema* (Johnson, 2007, p. 144) and is only one example of so-called embodied schemata that arise from the projections of the kinesthetic source domain to the domain of music (Barsalou, 1999; Lakoff, 1993; Lakoff & Johnson, 1980).

The concepts of line, curve, and path have a lot of operational power in this regard, even if they are used sometimes interchangeably. They can easily be depicted in a visual way, either in a one-dimensional, two-dimensional, or three-dimensional space. Things become more complicated, however, when we conceive of the “materiality” of sound in more than three dimensions and when we depart from the conception of a musical object as a volume that resembles a solid. Conceiving of music in “rheological” terms, as vibrating sounding energy that “flows” over time—from the Ancient Greek ῥεῖν (rheîn = to flow, stream, run), it is possible to conceive of it as occupying a position on the continuum between liquid and solid (see my target article, Reybrouck, 2025 for in-depth discussion). Thom’s distinction between static and metabolic forms is quite illuminating here: a classic example of a static form is a solid object, such as a pebble; examples of metabolic forms on the contrary are defined mainly by their kinematics; they include a jet of water, a plume of smoke, a flame, and living beings. The distinction, however, is an idealization that is difficult to maintain as most static forms are merely pseudo-static, if we examine at a sufficiently fine scale the underlying phenomena that ensure its stability (Thom, 1972, p. 109).

Music, as a dynamic art, is never totally petrified but should always be described in terms of dynamic conditions of structural changes, such as fluidity, elasticity, and plasticity. It brings us to the concept of transformation in the sense that the material form of the music at a specific moment can change its shape and evolve into another, thus changing its morphology. The concept of *morphodynamics* is looming here, of course, but the concept of *morphogenesis* might convey this even better by emphasizing the origination of the change in morphology.

**Figure 8:**
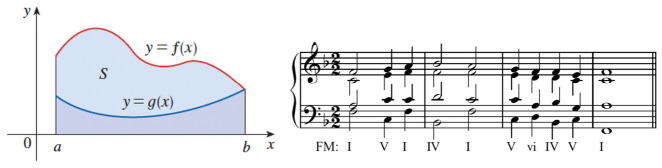
Example of an area of a bounded region between two curves (left) (AI-generated) and a four-voice texture in a musical score (Retrieved from https://itoldya420.getarchive.net/media/four-voice-texture-in-genevan-psalter-da7233?action=download&size=original (Copyright © Public domain).

Questions can be raised however regarding the concreteness of the transformations that underlie these changes of shape. The above-mentioned concept of sound tracking seems to imply a strong perceptual bonding with the sounding music, but there is also what Thom coins as morphogenesis *in abstracto.* Let’s take a somewhat uncommon example to sketch our problem. In the case of reading a mathematics textbook, there are multiple figures that are depicted graphically and that are also explained in the main text. Figure 8 depicts an example of finding the area of the region S bounded by two curves *y* = *f*(*x*), *y* = *g*(*x*) and the vertical lines *x* = *a*, *x* = *b.* The easiest way is to keep looking at the drawing while reading the text, but experienced mathematicians can even read the text and image the content without looking at the depicted curves, somewhat analogous to skilled music performers or conductors who can read the score and imagine how the printed notes sound without actually producing them. The latter is a process of sonification of printed symbols at a virtual level of imagery with many degrees of freedom as to the actual sounding characteristics. Just as the concrete trajectories of the curves of the functions f and g do not really matter for the understanding of the intuition of an area between them, it is arguable to conceive of a kind of inverted sonification and to transform the heard music to some kind of virtual shape in a virtual space. The musical notation example can be read in a similar way with the notes sounding in imagination without real perceptual bonding.

It is thus possible to formulate a purely geometric theory of morphogenesis in the abstract, independent of the substrate of forms and the nature of the forces that create them. The idea, however, may meet with resistance, especially for experimentalists who are accustomed to working with real-world data and who are constantly grappling with a reality that resists them (Thom, 1972, p. 25). The same applies to score reading in music, which is highly visuocentric and rather narrowly restricted to the domain of pitch. Questions can be raised about the psychological reality and ecological validity of score notation, but its value as a mediational tool between conception, performing and listening is absolutely beyond dispute. What I argue for in this article, however, is a broadening of scope that tries to materialize and spatialize music not solely in terms of pitch but also in a more holistic and syncretic way by equating it with a kind of virtual sonorous mass that is subject to ongoing and time-varying transformations. It once again raises the question of the bearers of form with pitch and time still being the “morphophoric” medium that is most analogous to the cognitive structural constraints that govern visual-spatial representation (Attneave, 1972; Attneave & Olson, 1971; Kubovy & Pomerantz, 1981; Shepard, 1981, 1982a, 1982b). It also means that musical meaningful objects such as melodies and chords are among the closest auditory analogs of visual shapes because these objects preserve their structure under rigid transformations within the media that function as the bearers of form (Shepard, 1982a). Pitch, however, is only one of the possible psychophysical continua to have such morphophoric properties. It may be interesting, therefore, to explore whether other continua such as, e.g., loudness or timbre could be considered as well (Attneave & Olson, 1971, p. 165).

### 3.4. Transformation and Invariance

**Figure 9:**
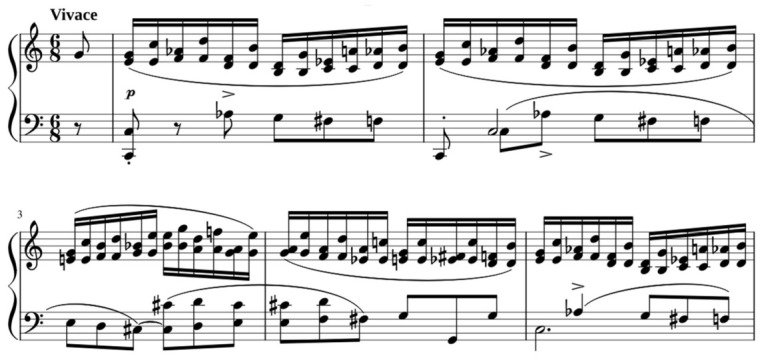
Example of the first 5 bars of Chopin’s Nocturne No 7, Op. 10 (Copyright © public domain). The patterns in the top staff repeat themselves either unchanged or transposed.

Psychophysical media are those in which patterns occur, which means that invariances are reflected in transposition behavior. The latter means, in the simplest case, that two perceptually equivalent patterns differ only by an additive constant. There are multiple examples of such transpositions in music, especially in piano music, with melodic or harmonic patterns that repeat themselves at higher or lower pitch levels, while maintaining mainly the same melodic shape (see Hofstadter, 1986 for an overview). The technique is used as a foundational tool for the development of musical ideas and to create a sense of direction—either in an ascending or descending way. Figure 9 depicts an example. It shows the first 5 bars of Chopin’s Nocturne, Op. 10, No 7. The patterns in the right hand (upper staff) have the same overall shape but are repeated at the same or different pitch level thus creating a sense of forward movement that is articulated also explicitly by using phrasing slurs. Close inspection of the patterns, however, shows that the intervallic distances between the distinct notes is not always preserved in a strict way.

Proceeding on the lines sketched above, it is tempting to conceive of this patterning technique in terms of *topological transformations* and even the mathematical intuitions underlying “linear algebra” and “group theory” may come to mind here. It should also be mentioned that the mathematical theory of transformation is part of group theory, which is quite important in modern mathematics. A distinction should be made, however, between substitution and transformation groups, in the sense that substitution groups are discontinuous—with abrupt changes between the figures—while transformation groups are continuous with infinitely small differences between the successive transformations (d’Arcy Thompson, 1917/1942, p. 1032). This technique of transformation, according to d’Arcy Thompson, is an important part of morphological studies, which aim at “comparing” related forms rather than providing a precise description of each of them. As such, it is possible to comprehend the deformation of a complicated figure while leaving the figure itself unanalyzed and undefined. What matters in the first place, is the process of comparison and of recognizing in one form a definite permutation of deformation of another, apart from a precise and adequate understanding of the original form (d’Arcy Thompson, 1917/1942, p. 1032).

There is no space to go into details here. It may suffice, for now, to think of the elementary use of the method *of coordinate transformations* that underlies the broader theory of transformations. It is an easy method for changing the numerical coordinates of a point from one reference system to another, resulting in a rather passive operation where the physical position of an object remains largely fixed and unchanged, but where its description changes due to been seen from a distinct vantage point.

**Figure 10:**
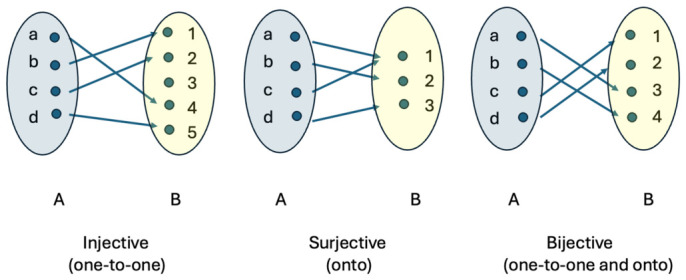
Schematic depiction of the relation between two sets in terms of injective, surjective, and bijective functions.

It is not difficult to think in terms of *set theory* in this regard, and to conceive of a set of elements (A) that are all moved together to another set (B), as is the case in a bijective function with a one-to-one correspondence between the elements of two sets. Musical transformations, however, are not always one-to-one, which means that the whole conceptual framework of injective, surjective, and bijective functions can be used as a source of inspiration (see Figure 10).

It is possible to focus on the sets themselves—and on their elements, taken together—but also on the transition between the sets. This holds especially for music as a temporal art, where the simultaneity of the visual depiction is outweighed by a more discursive way of processing. As Cassirer puts it, the elements of time are such only insofar as consciousness passes through them, and in this passing through—this “discursus”—they take on the characteristic form of the concept of time itself (Cassirer, 1922/1977, p. 170).

Much more can be said about the transformations of patterns into each other but that would take us too far. Even the concept of pattern can be discussed, but a broader conception of ongoing transformations of the sonorous mass of the music over time opens a lot of perspectives for musical sense-making in a real-time listening situation. Multiple transformations are possible in the development of musical themes, with some striking parallels with *regressive distortions* of percepts in dreams and imagery. Fisher distinguishes several of them: displacement of percepts or parts of percepts, condensation and fusion of percepts, fragmentation of percepts, rotational displacement, such as mirroring or 90° rotation, change in size, duplication and multiplication of the perception, loss of perspective relationships, retention of percepts, loss of the object’s meaning and replacement by another meaning, and change in form (Fisher, 1956; see also S. Friedman, 1960).

The analogy may seem a bit far-fetched but delving more deeply into musical transformations may be quite revealing in the sense that identical transformations—meaning that the figure remains unchanged—are the exception rather than the rule. A musical work, ideally, exhibits an internal coherence that can be regarded as a dynamic structure, whose elements are connected to one another in a systematic manner. As a rule, they involve relationships of affinity or temporal adjacency. According to Reti, structural design unfolds within the tension between “full identity” and “complex non-relationship,” with four primary possibilities: (i) “imitation” or the literal repetition of forms, either directly or through inversion, mirroring, etc.; (ii) “variation” or the alteration of forms in a gradual yet clearly recognizable manner; (iii) “transformation” or the creation of essentially new forms while retaining the original substance; and (iv) “indirect affinity” or the creation of an affinity between independent forms based on secondary characteristics (Reti, 1961, p. 238).

**Figure 11:**
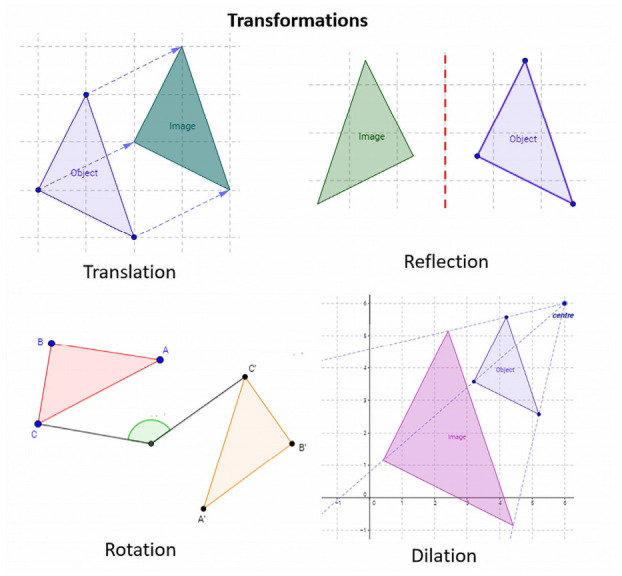
Schematic depiction of geometric transformations by sliding, flipping, rotating or resizing an original object. The image of the object can be congruent (translation, reflection, rotation) or similar (dilation) (ID 130598728, Copyright © Aleksandr Lysenko|Dreamstime.com, public domain).

It makes sense to conceive of these relationships in terms of *mental operations* that adhere to some extent to the foundations of geometry. The concept of congruent figures is essential in this regard: congruent figures are such that they can be superimposed on one another or made to coincide by translation, which is a geometric transformation that slides a shape over a specific distance in a given direction, without rotating, resizing of flipping it. As such it is a kind of rigid motion that retains the size, shape and orientation of an object while changing its position on a coordinate plane. Figure 11 depicts a schematic example of the basic transformations of objects into images of them, which are either isometric because the size of the image is the same as that of the object (translation, reflection, and rotation), or not isometric because the size of object and image are not the same (dilation). The examples, however, are ideal cases that do not always correspond to reality as it is quite difficult to move a figure without distorting it so that it remains unchanged. The idea of (mental) displacement of a figure, however, is quite illuminating.

Summarizing a little, there are two elementary classes of transformations: linear operations on a given dimension or group of dimensions, as in the case of translation, expansion, rotation, etc. but without changing the structure of the figure or the pattern, and those modifications that change the structure of the figure itself (McAdams, 1989). A major question in this regard is which description is needed to support the perception of shape and its transformations. Or put in other terms: which geometry is most suitable in this regard. The most familiar one is the Euclidean geometry in which objects that are metrically equivalent—by having the same measurement—are considered to have the same shape. Such “shape invariance” can then be summarized as a distance relationship that maintains the same distance between two points on a transformed object as between two corresponding points on the original object. Examples of such distance-preserving transformations are rotation, translation, and reflection (Michaels & Carello, 1981, p. 31). The reference to geometry is helpful here as a lot has happened in its recent history and the way it can be defined as formulated by Klein in his Erlangen program in an attempt to define geometry by the group of transformations that leave invariant certain properties of a space (Klein, 1872/1893).

It also makes sense to refer to Poincaré’s conceptions about our experience of space in this regard and to apply them to the act of listening (see below). By arguing that it is possible to pass from the *set of impressions* A to the set B, it is possible to move in two different ways, either involuntarily and without experiencing muscular sensations, or voluntarily and with muscular sensations. The former way implies that the object in question moves while the latter way lets the object immobile and lets us move so that the object is in relative movement in relation to us (Poincaré, 1907, p. 77). The idea is somewhat related to transformational theories of perception and judgment, which revolve around transformational mappings and the assumption that the perception of a static object depends heavily on the implicit representation of the possible transformations of that object (Shepard, 1981).

Poincaré’s conceptions met with skepticism at first, but they are currently gaining traction due to the emergence of the embodied and enactive turn in cognitive science. His conception of solid bodies and their relation to geometry is quite challenging for our approach: solid bodies are those among the bodies that surround us that frequently undergo displacements and that can be corrected by a corresponding movement of our body. Other objects with variable shape undergo such changes in position without changes in shape only exceptionally. It is thus possible to distinguish *deformations* from other changes of state in the sense that in deformations each element undergoes a simple change of position which can be corrected, but the change that is undergone by the body as a whole is more profound and cannot be corrected by a corresponding movement (Poincaré, 1907, pp. 79–80). These insights are quite inspiring when it comes to apply them to the kinematics of music listening and the related conception of phoronomic listening as explained below. Before proceeding further on these lines, however, it‘s worth recalling the above-mentioned distinction between two kinds of movement: those that are attributed to external objects and that proceed involuntary and without muscular sensations (external changes) and those that are attributed to our body (internal changes). Both are reciprocal and can correct each other, but this applies especially to displacements (Poincaré, 1907, p. 82).

## 4. Phoronomy and the Kinematics of Music Listening

To finally get to the major point of this article, it is not difficult to conceive of music in terms of movement of a virtual object over time and to “trace,” as it were, its actual unfolding in a virtual space. Such an ongoing and continuous sound tracking may have some resemblance with the drawing of a line. I therefore recently coined the term *phoronomic listening* in this regard (Reybrouck, 2025), with a reference to Kant, who introduced the term for the study and composition of motions of bodies without considering their generating forces (Kant, 1786/1900 and M. Friedman, 2013 for a critical discussion). In a somewhat derived sense, the term can be understood as the dynamic aspect of the metaphorical projection of a line, which can be generalized more broadly to the generation of a curve. Such a curve can then be regarded as engendered by the movement of a point, subjected to some lawful constraints. It exemplifies the role of the embodied prehension of a geometric figure which is the product of the combination of certain elements of construction—such as points and lines—and movements, echoing to some extent the method of *genetic definition* of the ancient Greeks, which corresponds to the “dynamic” vision on axiomatics in geometry (Defrise, n.d., p. 30). The concept is not (yet) commonly used in music-related studies, but the term is intuitively appealing, especially when trying to translate the felt experience of sounding music in terms of manifest or imagined movements.

### 4.1. The Concept of Phoronomy as a Pure Doctrine of Motion

Phoronomy—from the Ancient Greek φέρειν (ferein = to carry along) and νόμος (nomos = law or rule)—is a philosophical theory of motion, introduced by Kant, who defined it as a “pure doctrine of motion.” The term refers to the first chapter of his *Metaphysical Foundations of Natural Science* (Kant, 1786/1900), which is a dense and difficult-to-digest little book that provides the metaphysical foundations of natural science, more in particular Newton’s physics. It contains four chapters—phoronomy, dynamics, mechanics, and phenomenology—each of them adding new aspects to the concept of motion. They imply each other with an increasing level of hierarchy, which means that the latter presuppose the former (Kant, 1786/1900 see also Grusea, 2022; Vuillemin, 1955; Walker, 1995). As to the first—phoronomy—its metaphysical foundation is built on Kant’s mathematical theory of cognition, which states that the composition of motion rests on the construction of extended intervals in space and time. Matter, in this view, is what is mobile in space; the space which is also mobile itself is called the relative space; and the space in which all movement must ultimately be thought of is absolutely immobile and is called the absolute space (see Sutherland, 2014). Space and movement, therefore, are always relative.

Kant’s writings are cryptic and not easy to understand. He also does not clearly indicate whether his “theory of motion” adheres to the first (phoronomy) or to the third (mechanics) chapter of his Foundations. The latter considers the moving insofar as it has a moving force. A general theory of motion, on the contrary, should also include kinematic movements as a description of the change of certain changes of a state in general, not only those that presuppose a force. As such, it should be a pure theory of magnitude of motion, independent of any empirical properties. Kant’s approach is thus an attempt to introduce time into geometry in the sense that matter is known through its property of mobility.

**Figure 12:**
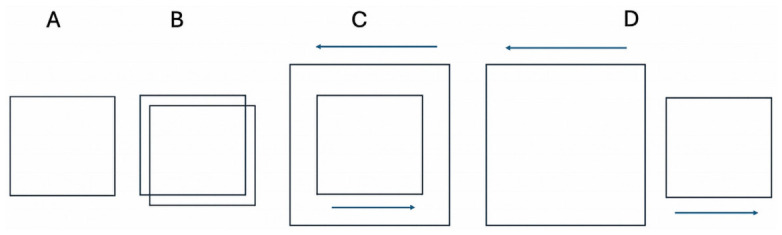
Example of the idea of doubling of space with superposition of spaces and their relative movement (Copyright © Slightly modified and adapted from Grusea (2022)).

Kant’s theory of mathematical cognition is hard to grasp, but it has been instrumental in relating his central concepts of phoronomy to the concept of the spectator and his/her ways of constructing motion. Especially his *method of doubling space* has triggered much attention afterwards (Kant, 1786/1900, p. 48; see also Vuillemin, 1955). The basic idea is quite simple: every movement that can be an object of possible experience can be regarded as the movement of our body in a still or resting space but also as the body in rest with movement of the space. It highlights the role of *self-localization* of the spectator and the relativity of movement (Grusea, 2022), which means that it is possible to get two spaces from a single geometric. Perceived motion, therefore, is relative: there is the motion of an object within a frame of reference and the motion of that frame itself within a larger frame. As such, there is no absolute, fixed reference point for motion in experience (Stan, 2022). The idea is illustrated rather intuitively in Figure 12 where the superposition and relative movement of two spaces (surfaces in this case) is shown. There is first a doubling of space from Figure 12A to Figure 12B, and then the start of a relative movement at Figure 12C with movement of both spaces in relation to each other in Figure 12D.

It would take us too far afield to delve in Kant’s conceptions of the intuitive origins of spatial representation (see Smyth, 2014; Callanan, 2014). Two notions, however, deserve attention: the notion of *self-localization* of the perceiving subject and the *relativity of movement*. Both are related to the structure of the doubling of space and the related question whether this must be carried out by a perceiving subject. Kant’s answer lies in the relation of the central concepts of phoronomy to the concept of the spectator: when a body moves in relative space we can conceive of the space as resting and the body as moving, but we can also conceive of the body as resting and the space moving in the opposite direction at the same speed. Or stated more generally: every movement, as an object of possible experience, can be regarded as the movement of the body in a still or resting space but also as that body being in a state of rest in a moving space (Kant, 1786/1900, p. 487). The principle of phoronomy, therefore, is based on the relativity of movements in space and the self-positioning of the subject, which makes the concepts of direction and speed possible.

### 4.2. The Deictic Approach and Its Relation to Phoronomy

There is further a tension between the concept of three-dimensional space, which is given as it were, and that of a generated space that is structured by directions. Both conceptions differ from each other, but they can both be considered as being mobile. A first step to distinguish them is the introduction of a *zero point* in uniform space as a spatiotemporal reference system. In coordinate systems, such a zero point is determined by convention, but in indexical or egocentric reference systems it is automatically provided by the position of the perceiving subject (Keil, 2011). The establishment of such a zero point, however, is not merely the introduction of a neutral geometric point, but as an “I am here”-point it also means that the designation of “I” and “here” coincides with the bodily self rather than being a point among points (Kaulbach, 1960, p. 91).

It is this bodily I itself, which expands, and which makes it possible to come to determinations such as left/right, above/below, and front/back, which ultimately imply internal determinations with self-localization as a starting point. This opens a different spatiality, which has both an internal and external structure, created by the act of localization, but which is also determined by the given three-dimensionality of space (Kant, 1768, p. 378). Self-localization, in Kant’s view, is thus a basic condition of his phoronomy. It entails the doubling of space and opens the possibility that the spaces that are distinguished move in different directions and that the direction in which we move can only be known if we already know the location where we are (Grusea, 2022, p. 293).

Directions, such as left/right, top/bottom, and front/back, do also not merge into a homogenous extension of space. They entail internal determinations of space, which means that self-localization opens up a different spatiality: on the one hand it has an internal structure with directions that are not objectively given, but generated by the act of localizing; on the other hand there is the body that is always determined by the three-dimensionality of space (Woelert, 2007). It is an approach that fits to some extent with the *deictic approach*, which dates back to Bühler’s distinction between “pointing words” like “this/that,” “I/you,” “here/there,” and “naming words” in general. It thus draws an explicit analogy between gestural and linguistic means for showing direction and place (Bühler, 1934/1965, 1982; see also Kita, 2003 for a broader overview).

**Figure 13:**
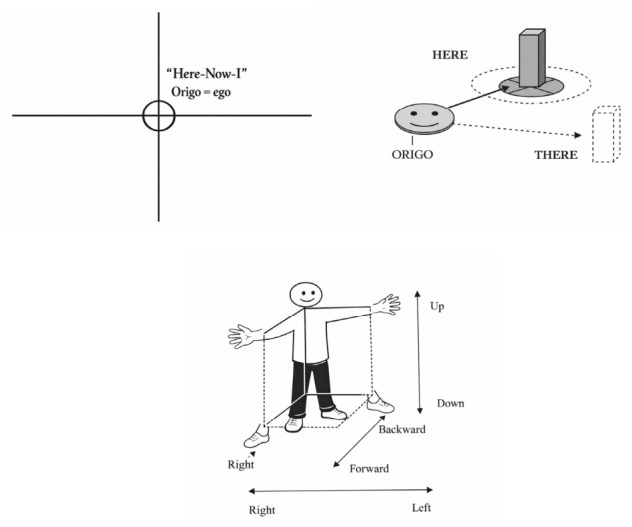
Schematic for the introduction of a zero-point as a mere point in space (upper left), as a spatial anchor point (upper right), and as a blurring of self-localization with horizontal and vertical directions as left/right, above/below, and front/back (Copyright © AI-generated).

Deictic expressions thus refer to the “field of pointing”—Bühler’s *deictic field*—rather than to the “symbolic field of meaning, and make it possible to take an indexical—from the Latin index for index finger—approach to sense-making (Hanks, 2005; Lyons, 1982). It is thus possible to conceive of a transition from a mere localization of a *zero-point* or *origo* in space to the identification of the spectator with this origo and to use this self-localization as the starting point for experiencing directionality in space. It allows the bodily self to expand itself and to conceive of determinations such as left/right, above/below, and front/back. The latter, then, ultimately imply internal determinations which take self-localization as a starting point (Grusea, 2022). The whole transition is depicted schematically in Figure 13.

**Figure 14:**
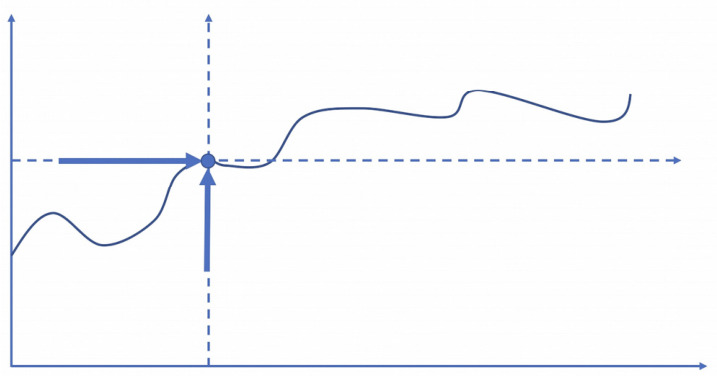
Phoronomic interpretation of a curve as the result of two perpendicular movements along the two axes of the figure (Reproduced without modification from Reybrouck, 2025, Copyright © 2025, Creative Common Atribution (CC BY) license).

The deictic approach can be seamlessly applied to the realm of music. It suffices simply to adopt a dynamic approach that captures the temporal unfolding of the articulation of the sound (Reybrouck, 2009, 2015). This means that listeners can mentally point to the sonorous unfolding to perceive sound events as things that are on the one hand logically discrete and isolable but that can also be perceived as continuous gestures (Serafine, 1988, p. 75; see also Gjerdingen, 1994; Godøy, 2006; N. Todd, 1992; P. Todd, 1999). It is then possible to define the sonorous unfolding as a function of time, which, in an intuitive representation as a curve, should be seen as a conditional equation with two unknowns or variables. To function as a continuous function, however, this should entail that an arbitrary change of the values of one of the variables (the abscissa, functioning as the independent variable) should simultaneously change the values of the other variable (the ordinate, functioning as the dependent variable). Such secondary school mathematics may seem to be trivial, but it provides a very intuitive conception of the *genesis* of a curve in terms of motion, which means that it can also be described in “phoronomic” terms. Figure 14 depicts an intuitive example. It shows how the simultaneous perpendicular movements along the two axes that correspond to the two unknowns determine points that can be connected to plot a curve (Colerus, 1935, p. 313). The motion of both variables, however, is not always strictly uniform, thus resulting in a straight line. In reality, that motion is mostly arbitrary and non-uniform, which means that the resulting curve can draw an irregular “path” of a point from some beginning to some end.

The analogy with a melodic line is obvious here, though the selection of pitch as the only morphophonemic feature of music is clearly too limited, as mentioned already above. More fruitful is an approach that reduces the spectromorphological configuration at every moment to a point in virtual space that follows a specific path through time. The points, in that view, should then be considered as abstractions for collections of constitutive elements. It is an approach that is commonly used in *mathematical category theory* (Lawvere & Schanuel, 2009; Mac Lane, 1998). The technicalities of this approach, however, are too complex to be dealt with in this introductory paper, but the key message is that not the contents of the collections, but the relations between these collections are of primary importance. It is thus tempting to conceive of the listening process as a movement through time with listeners who may align their attentional focus with the music, considered as a kind of virtual matter that flows and changes its shape in an ongoing way. The possible application of category theory to the realm of music, however, is still in an embryonic stage (see Mannone, 2019; Mazzola, 2002). Much is to be expected here from the weakest kind of geometry, namely *topology*, which deals with the most liberal concept of shape, and which deals with the recognition of invariance on qualitative rather than quantitative grounds. This also means that the abstractness of the topological invariants implies a certain abstractness of the perceptual information that underlies their perceptual constancy (Michaels & Carello, 1981, p. 36). It also brings us back to Poincaré, who, as an avid reader of Kant (see Folina, 1992; Godlove, 2009) and being the founder of algebraic topology, adapted, modified, and defended key elements of the Kantian framework to fit non-Euclidean geometries and advanced mathematical developments of the 19th and 20th centuries.

### 4.3. Real-Time Listening and the Consumption of Sound

The idea that listeners can mentally point to the sonorous unfolding and simultaneously perceive it as a continuous gesture is not merely a poetic metaphor. It revitalizes certain older movements of the 1930s, which have recently received empirical support from the cognitive sciences and the more narrowly defined field of enactive cognition (Reybrouck, 2025). Summarizing a little, it can be stated that the gestural dynamic reconstruction of the music brings together continuous and discrete processing of the sound (Gritten & King, 2013). It implies the succession of multiple focal points, echoing the distinction between *processual predication* and *episodic nominalization*, with the former following the temporal evolution of a situation—involving a continuous series of points in conceived time (its temporal profile)—and the latter referring to just a single instance of the process—as a thing or event that can be characterized as a “bounded region” in some domain (Langacker, 1987, pp. 191 and 244).

Music listening, ideally, should imply both modes of processing. The dynamic character of music as a temporal art, however, entails a processual approach with specific attentional dynamics, transforming listening into an *action verb*. Any conscious engagement with music should therefore be viewed against the backdrop of what is present in the listener’s consciousness. Contrary to a geometric figure which is present at once, a melodic figure or some broader sonorous unfolding requires a successive presentation of its constituting elements that dictates a specific course upon our actions. This means that we must wait until the temporal unfolding is complete before it can be considered in and of itself or being recognized as such. It led Francès to name the moment in the unfolding when recognition takes place as a result of the summation of preceding element the *point of condensation* (Francès, 1958, p. 247).

There is, as such, an aspect in the perception of a temporal unfolding, which Dewey had called the *consummatory phase* of experience, and which is intervening in its course as well as occurring at the end. It also means that there is a compression from accumulation in time, be it in hearing music, reading a poem or seeing a drama enacted. No work of art can be perceived in one instant because simultaneity does not allow for conservation and increase in tension and ultimately for the release and unfolding that gives volume to a work of art. The more of what precedes is condensed, the richer is the present perception and the more intense its forward impulsion. Or put in other terms: the release of contained materials as they unroll gives the subsequent a wider span (Dewey, 1934/1958, pp. 138 and 183).

## 5. Conclusions and Perspectives

The current article arose from a feeling of dissatisfaction with a lot of traditional music research, which is symbol-based—with a major emphasis on score analysis—rather than behavioral. Such research mostly considers music as an artifact that is analyzed outside the time of sounding. Without having the intention to detract from the benefits of the score as a mediating tool for performing, I have a feeling—shared among many scholars—that something is missing when it comes to describe the musical experience by the listener. This holds in particular for the description of the temporal and energetic dynamics that make music a living thing rather than a petrified artifact.

This article, therefore, was a follow-up of a preceding article where I defined music as a *fluidum* (Reybrouck, 2025). While this target article was both theoretically and empirically grounded, the current article is more like a hypothesis and theory article with the aim to bring together some older insights from Kantian philosophy, some mathematical intuitions about the relations between space and time, the theoretical framework from morphodynamics and topological transformation, and possible applications within the broader field of embodied and enactive cognition as applied to music. Rather than surfing on the trendy issues of the day, it is an attempt to provide some “foundational work” by raising questions rather than giving answers (see Section 1). In doing this I relied on several ways of grounding, with, among others, some key analogies such as river dynamics and virtual trajectories, which were used in a rather metaphorical sense. Other constructs, such as density curves and morphodynamic transformations lend themselves better to analytical descriptions, even if they are used in a fairly broad sense. The ultimate goal, however, was to come to some proposed psychological mechanisms to deal with music in behavioral terms. The major focus on *phoronomic listening* and its close connections to sound tracking is above all an exercise in attentional dynamics that is externalized in terms of behavioral analogies as the tracing of a curve. This can be either manifest or virtual, which makes it difficult to subject it to empirical testing. It is important therefore, to give the initial claims that were developed in this article some more empirical weight by changing them in testable statements:

- music can be represented as a temporally unfolding virtual object;
- active listening involves sensorimotor tracking of this object;
- music listening can be reframed as a dynamic, real-time kinetic gesture rather than the passive tracking of an acoustic artifact;
- defining listening as an action verb offers a behavioral alternative to static, score-bound music analysis;
- phoronomic listening can be externalized through gesture or trajectory-based visualization;
- all these claims can be tested through movement, physiological or neurocognitive measures.

Some of these statements can rather easily be translated into empirical research. This holds in particular for sensorimotor tracking, motion-caption research, and neural correlates of music listening with many studies that have already been published in journals outside the field of musicology. It makes sense, therefore, to conceive of a kind of “behavioral turn” in the study of music and dealing with music, with a change in focus from music-analytical descriptions to more observable listening behaviors, such as gesture, movement tracking, motor synchronization, attentional tracking, imagery, physiological entrainment, or subjective reports.

Taken together, this article tried to provide an alternative but less obvious way to make sense of music in a real-time listening situation. The insights and ideas that were collected from diverging disciplines have in common that music can induce a kind of internally felt motion—i.e., ideomotor simulation or resonance—not merely limited to recognizing or tapping the beat, but aimed at uncovering the explanatory mechanisms for resonating with the minute time-varying temporal and dynamic modulations of the temporal unfolding. By providing some strong intuitions from pioneers—and even founding fathers—within and outside the field of music research, which mostly are cited rather than being read, especially when it concerns older contributions, it was the aim of this article to open new perspectives for future research. There are currently virtually no studies about phoronomy and rheology as applied to music, though the analogy between music and flowing energy may seem to be quite obvious. While the target article focused mainly on the “music” as flowing energy—music as fluidum—the current article focuses more strongly on the “listener,” who might be invited to move along with the music at a virtual level of ideomotor simulation. A possible shortcoming of this approach, however, could be its apparent limitation to the momentary grasping of the sound in successive now-moments that evolve in a continuous way over time, while simultaneously losing track of the overall big picture. The latter, however, cannot be built without putting together the smaller constitutive elements, which is another explanatory mechanism for musical sense-making. It refers to the synthesizing function of time. There is, as such, a dynamic tension between short-lived sounding phenomena, their concatenations, and the feeling of relational continuity with time as the substrate for a navigation trajectory through the sounding music. The elaboration of these complex relationships, however, falls outside the scope of the current article.

## Data Availability

No available data.

## References

[B1-behavsci-16-01092] Andrey V. (2025). What is calligraphy? Discover different types of writings. Artsper Magazine.

[B2-behavsci-16-01092] Ashby W. (1956). An introduction to cybernetics.

[B3-behavsci-16-01092] Attneave F., Melton A. W., Martin E. (1972). The representation of physical space. Coding processes in human memory.

[B4-behavsci-16-01092] Attneave F., Olson R. (1971). Pitch as a medium: A new approach to psychophysical scaling. The American Journal of Psychology.

[B5-behavsci-16-01092] Barsalou L. (1999). Perceptual symbols systems. Behaviorial & Brain Sciences.

[B6-behavsci-16-01092] Battey B. (2015). Towards a fluid audiovisual counterpoint. Ideas Sónicas.

[B7-behavsci-16-01092] Blow M. (2014). On the simultaneous perception of sound and three-dimensional objects. Ph.D. thesis.

[B8-behavsci-16-01092] Bonini L., Rotunno C., Arcuri E., Gallese V. (2022). Mirror neurons 30 years later: Implications and applications. Trends in Cognitive Sciences.

[B9-behavsci-16-01092] Brandner H. (2018). On the status quo of alexander Truslit’s tutorial of musical movement. Jahrbuch Musikpsychologie.

[B10-behavsci-16-01092] Burton D. (2011). The history of mathematics/an introduction.

[B11-behavsci-16-01092] Bühler K. (1965). Sprachtheorie: Die Darstellungsfunktion der Sprache.

[B12-behavsci-16-01092] Bühler K., Goodwin D. F., Jarvella R., Klein W. (1982). The deictic field of language and deictic words. Speech, place, and action. Studies in deixis and related topics.

[B13-behavsci-16-01092] Callanan J. (2014). Kant on the acquisition of geometrical concepts. Canadian Journal of Philosophy.

[B14-behavsci-16-01092] Cassirer E. (1977). Philosophie der symbolischen Formen. Erster Teil. Die Sprache.

[B15-behavsci-16-01092] Chemero T. (2009). Radical embodied cognitive science.

[B16-behavsci-16-01092] Chi T., Ru P., Shamma S. (2005). Multiresolution spectrotemporal analysis of complex sounds. Journal of the Acoustical Society of America.

[B17-behavsci-16-01092] Clark A., Chalmers D. (1998). The extended mind. Analyses.

[B18-behavsci-16-01092] Clarke E. (2005). Ways of listening. An ecological approach to the perception of musical meaning.

[B19-behavsci-16-01092] Clayton M., Post J. C. (2017). Time, gesture, and attention in a Khyāl performance. Ethnomusicology: A contemporary reader, volume II.

[B20-behavsci-16-01092] Clayton M., Sager R., Wil U. (2005). In time with the music: The concept of entrainment and its significance for Ethnomusicol. European Meetings in Ethnomusicology.

[B21-behavsci-16-01092] Cogan R. (1984). New images of musical sound.

[B22-behavsci-16-01092] Coker W. (1972). Music and meaning. A theoretical introduction to musical aesthetics.

[B23-behavsci-16-01092] Colerus E. (1935). From the point to the fourth dimension. Geometry for everyone.

[B24-behavsci-16-01092] Colombetti G. (2007). Enactive appraisal. Phenomenology and the Cognitive Sciences.

[B25-behavsci-16-01092] Colombetti G. (2014). The feeling body. Affective science meets the enactive mind.

[B26-behavsci-16-01092] Comte A. (1998). Cours de philosophie positive; I leçons 1 à 45.

[B27-behavsci-16-01092] Cox A. (2016). Music and embodied cognition: Listening, moving, feeling and thinking.

[B28-behavsci-16-01092] d’Arcy Thompson W. (1942). On growth and form. Vol. I and II.

[B29-behavsci-16-01092] Defrise P. (n.d.). Visages de la mathématique.

[B30-behavsci-16-01092] Dewey J. (1958). Art as experience.

[B31-behavsci-16-01092] Di Iorio F., Sarti A. (2024). Jean Petitot’s new enlightenment. Morphology, neurogeometry, semiotics. A festschrift in honor of Jean Petitot‘s 80th birthday.

[B32-behavsci-16-01092] Di Paolo E., Rohde M., De Jaegher H., Stewart J., Gapenne O., Di Paolo E. (2010). Horizons for the enactive mind: Values, social interaction, and play. Enaction: Toward a new paradigm for cognitive science.

[B33-behavsci-16-01092] Fatone G., Clayton M., Leante L., Gritten A., King (2011). Imagery, melody and gesture in cross-cultural perspective. New perspectives on music and gesture.

[B34-behavsci-16-01092] Fisher C. (1956). Dreams, images, and perception. Journal of the American Psychoanalytic Association.

[B35-behavsci-16-01092] Folina J., Folina J. (1992). Introduction to Poincaré’s theory of the synthetic a priori. Poincaré and the Philosophy of Mathematics.

[B37-behavsci-16-01092] Franceschelli S., Sarti A. (2024). The delicate frontier between schematism and reflection. Morphology, neurogeometry, semiotics. A festschrift in honor of Jean Petitot‘s 80th birthday.

[B36-behavsci-16-01092] Francès R. (1958). La perception de la musique.

[B38-behavsci-16-01092] Friedman M. (2013). Kant’s construction of nature: A reading of the metaphysical foundations of natural science.

[B39-behavsci-16-01092] Friedman S. (1960). One aspect of the structure of music. A study of regressive transformations of musical themes. Journal of Psychoanalytic Association.

[B40-behavsci-16-01092] Froese T., Gershenson C., Rosenblueth D. (2013). The dynamically extended mind. A minimal modeling case study. arXiv.

[B41-behavsci-16-01092] Galantucci B., Fowler C., Turvey M. (2006). The motor theory of speech perception reviewed. Psychonomic Bulletin and Review.

[B42-behavsci-16-01092] Gallagher S., Zahavi D. (2008). The phenomenological mind. An introduction to philosophy of mind and cognitive science.

[B43-behavsci-16-01092] Gallese V. (2005). Embodied simulation: From neurons to phenomenal experience. Phenomenology and the Cognitive Sciences.

[B44-behavsci-16-01092] Gallese V., Eagle M., Migone P. (2007). Intentional attunement: Mirror neurons and the neural underpinnings of interpersonal relations. Journal of the American Psychoanalytic Association 55.

[B45-behavsci-16-01092] Gallese V., Goldman A. (1998). Mirror neurons and the simulation theory of mind-reading. Trends in Cognitive Science.

[B46-behavsci-16-01092] Gibbs R. (2010). Embodiment and cognitive science.

[B47-behavsci-16-01092] Gjerdingen R. (1994). Apparent motion in music. Music Perception.

[B48-behavsci-16-01092] Godlove T. (2009). Poincaré, Kant, and the scope of mathematical intuition. The Review of Metaphysics.

[B49-behavsci-16-01092] Godøy R. I. (2006). Gestural-sonorous objects: Embodied extensions of Schaeffer’s conceptual apparatus. Organised Sound.

[B50-behavsci-16-01092] Godøy R. I., Leman M. (2010). Musical gestures: Sound, movement, and meaning.

[B51-behavsci-16-01092] Gritten A., King E. (2013). New perspectives on music and gesture.

[B52-behavsci-16-01092] Grusea D. (2022). Die Selbstlokalisierung als Grundlage der Kantischen Phoronomie. Revue Roumaine de Philosophie.

[B53-behavsci-16-01092] Hanks W. (2005). Explorations in the deictic field?. Current Anthropology.

[B54-behavsci-16-01092] Hatten R. (2004). Interpreting musical gestures, topics, and tropes. Mozart, Beethoven, Schubert.

[B55-behavsci-16-01092] He L., Wang H., Yiwen Bian Y., Bhatia S. (2025). Information sampling and Bayesian belief formation in statistical judgment. Proceedings of the New York Academy of Sciences of the United States of America.

[B56-behavsci-16-01092] Henry M. (1963a). L’Essence de la manifestation (Tome premier).

[B57-behavsci-16-01092] Henry M. (1963b). L’Essence de la manifestation (Tome second).

[B58-behavsci-16-01092] Henry M. (1965). Philosophie et phénoménologie du corps.

[B59-behavsci-16-01092] Hofstadter D. (1986). Metamagical themas: Questing for the essence of mind and pattern.

[B60-behavsci-16-01092] Homer S., Harley N., Wiggins G. (2024). Contrast information dynamics: A novel information measure for cognitive modelling. Entropy.

[B61-behavsci-16-01092] Husserl E. (1989). Ideas pertaining to a pure phenomenology and to a phenomenological philosophy, second book.

[B62-behavsci-16-01092] Hutto D., Myin E. (2013). Radicalizing enactivism. Basic minds without content.

[B63-behavsci-16-01092] Jackendoff R. (1987). Consciousness and the computational mind.

[B64-behavsci-16-01092] James W., McDermott J. (1968). The writings of William James. A comprehensive edition.

[B65-behavsci-16-01092] Johnson M. (2007). The meaning of the body. Aesthetics of human understanding.

[B66-behavsci-16-01092] Kant I. (1768). Von dem ersten grunde des unterschiedes der gegenden im raume.

[B67-behavsci-16-01092] Kant I. (1900). Metaphysischen Anfangsgründe der Naturwissenschaft. Kants gesammelte Schriften, Band 4 *(Königlich Preußische Akademie der Wissenschaften)*.

[B68-behavsci-16-01092] Katz V. (2009). A history of mathematics: An introduction.

[B69-behavsci-16-01092] Keil G., Carson S., Knowles J., Myskja B. (2011). Ich bin jetzt hier—aber wo ist das?. Kant: Here, now and how. Essays in honour of Truls Wyller.

[B70-behavsci-16-01092] Kenny A., Calhoun C., Solomon R. (1984). Action, emotion and will. What is an emotion. Classic readings in philosophical psychology.

[B71-behavsci-16-01092] Kent A., Vujakovic P. (2017). The routledge handbook of mapping and cartography.

[B72-behavsci-16-01092] Kita S. (2003). Pointing. Where language, culture, and cognition meet.

[B73-behavsci-16-01092] Klein F. (1893). Vergleichende Betrachtungen über neuere geometrische Forschungen. Mathematische Annalen.

[B74-behavsci-16-01092] Kubovy M., Pomerantz R. (1981). Perceptual organization.

[B75-behavsci-16-01092] Kurth E. (1920). Die romantische harmonik und ihre krise in wagners “Tristan”.

[B76-behavsci-16-01092] Kurth E. (1931). Musikpsychologie.

[B77-behavsci-16-01092] Lakoff G., Ortonyi A. (1993). The contemporary theory of metaphor. Metaphor and thought.

[B78-behavsci-16-01092] Lakoff G., Johnson M. (1980). Metaphors we live by.

[B79-behavsci-16-01092] Langacker R. (1987). Foundations of cognitive grammar.

[B80-behavsci-16-01092] Lawvere F., Schanuel S. (2009). Conceptual mathematics: A first introduction to categories.

[B81-behavsci-16-01092] Leante L. (2009). The lotus and the king: Imagery, gesture and meaning in a Hindustani Rāg. Ethnomusicology Forum.

[B82-behavsci-16-01092] Leman M. (2007). Embodied music cognition and mediation technology.

[B83-behavsci-16-01092] Lesaffre M., Leman M., Maes P.-J. (2017). The Routledge companion to embodied music interaction.

[B84-behavsci-16-01092] Livingstone P. (2009). Narrativity and knowledge. Journal of Aesthetics and Art Criticism.

[B85-behavsci-16-01092] Lyons J., Jarvella R., Klein W. (1982). Deixis and subjectivity: Loquor, ergo sum. Speech, place and action. Studies in deixis and related topics.

[B86-behavsci-16-01092] Mac Lane S. (1998). Categories for the working mathematician.

[B87-behavsci-16-01092] Maiese M. (2011). Embodiment, emotion, and cognition.

[B88-behavsci-16-01092] Mannone M. (2019). Mathematics, nature, art.

[B89-behavsci-16-01092] Mazzola G. (2002). The Topos of music. Geometric logic of concepts, theory, and performance.

[B90-behavsci-16-01092] McAdams S., McAdams S., Deliège I. (1989). Contraintes psychologiques sur les dimensions porteuses de la forme. La musique et les sciences cognitives.

[B91-behavsci-16-01092] Meelberg V. (2006). New sounds, new stories: Narrativity in contemporary music.

[B92-behavsci-16-01092] Meelberg V., Khannanov I., Ruditsa R. (2021). Thinking/feeling musical narrative. Proceedings of the worldwide music conference 2021.

[B93-behavsci-16-01092] Mehrabian A. (1996). Pleasure-arousal-dominance: A general framework for describing and measuring individual differences in temperament. Current Psychology.

[B94-behavsci-16-01092] Mehrabian A., Russell J. (1974). An approach to environmental psychology.

[B95-behavsci-16-01092] Menary R. (2010). The extended mind.

[B96-behavsci-16-01092] Merleau-Ponty M. (1981). Phénoménologie de la perception.

[B97-behavsci-16-01092] Michaels C., Carello C. (1981). Direct perception.

[B98-behavsci-16-01092] Molnar-Szakacs I., Overy K. (2006). Music and mirror neurons: From motion to “e”motion. Social Cognitive and Affective Neuroscience.

[B99-behavsci-16-01092] Newen A., de Bruin L., Gallagher S. (2018). Oxford handbook of 4E cognition.

[B100-behavsci-16-01092] Nikolopoulou K. (2023). Action verbs|definition, list & examples. scribbr.

[B101-behavsci-16-01092] Noë A. (2004). Action in perception.

[B102-behavsci-16-01092] Okoroigwe N., Nkemdirim V., Ojo O. (2025). Fluvial and geomorphological analysis of river dynamics and impact. International Journal of Science and Research Archive.

[B103-behavsci-16-01092] O’Regan J., Noë A. (2001). A sensorimotor account of vision and visual consciousness. Behavioral and Brain Sciences.

[B104-behavsci-16-01092] Overy K., Molnar-Szakacs I. (2009). Being together in time: Musical experience and the mirror neuron system. Music Perception.

[B105-behavsci-16-01092] Pantev C., Lappe C., Herholz S., Trainor L. (2009). Auditory-somatosensory integration and cortical plasticity in musical training. Annals of the New York Academy of Sciences.

[B106-behavsci-16-01092] Paschalidou S. (2022). Effort inference and prediction by acoustic and movement descriptors in interactions with imaginary objects during Dhrupad vocal improvisation. Wearable Tehnologies.

[B107-behavsci-16-01092] Paschalidou S. (2024). Technology-mediated Hindustani dhrupad music education: An ethnographic contribution to the 4E cognition perspective. Educational Sciences.

[B108-behavsci-16-01092] Petitot J. (1992). Physique du Sens.

[B109-behavsci-16-01092] Petitot J. (2009). Per un nuovo Illuminismo.

[B110-behavsci-16-01092] Piaget J. (1967). Biologie et connaissance. Essai sur les relations entre les régulations organiques et les processus cognitifs.

[B111-behavsci-16-01092] Plack C., Plack C., Fay R., Oxenham A., Popper A. (2005). Pitch perception models. Pitch. Neural coding and perception.

[B112-behavsci-16-01092] Poincaré H. (1907). La Science et l’hypothèse.

[B113-behavsci-16-01092] Pollard C. (2014). Merleau-Ponty and embodied cognitive science. Discipline Filosofiche.

[B114-behavsci-16-01092] Prinz W., Chater N., Hurley S. (2005). An ideomotor approach to imitation. Perspectives on imitation: From neuroscience to social science. Vol. 1: Mechanisms of imitation and imitation in animals.

[B115-behavsci-16-01092] Rahaim M. (2012). Musicking bodies: Gesture and voice in Hindustani music; Music/culture.

[B116-behavsci-16-01092] Ramati A. (2001). The hidden truth of creation: Newton’s method of fluxions. British Journal for the History of Science.

[B117-behavsci-16-01092] Repp B. (1993). Music as motion: A synopsis of Alexander Truslit’s (1938) Gestaltung und Bewegung in der Musik. Psychology of Music.

[B118-behavsci-16-01092] Reti R. (1961). The thematic process in music.

[B119-behavsci-16-01092] Reybrouck M., Godøy R. I., Jörgensen H. (2001). Musical imagery between sensory processing and ideomotor simulation. Musical imagery.

[B120-behavsci-16-01092] Reybrouck M. (2009). Similarity perception as a cognitive tool for musical sense-making: Deictic and ecological claims. Musicæ Scientiæ, Discussion Forum 4B.

[B121-behavsci-16-01092] Reybrouck M. (2015). Real-time listening and the act of mental pointing: Deictic and indexical claims. Mind, Music, and Language.

[B122-behavsci-16-01092] Reybrouck M. (2020). Music listening as adaptive behaviour: Enaction meets neuroscience. Journal of Interdisciplinary Music Studies.

[B123-behavsci-16-01092] Reybrouck M. (2021). Musical sense-making. Enaction, experience, and computation.

[B124-behavsci-16-01092] Reybrouck M. (2023). A dynamic interactive approach to music listening: The role of entrainment, attunement and resonance. Multimodal Technologies and Interaction.

[B125-behavsci-16-01092] Reybrouck M. (2025). Music as fluidum: A rheological approach to the materiality of sound as movement through time. Behavioral Sciences.

[B126-behavsci-16-01092] Rizzolatti G., Craighero L. (2004). The mirror-neuron system. Annual Review of Neuroscience.

[B127-behavsci-16-01092] Russell J., Mehrabian A. (1977). Evidence for a three-factor theory of emotions. Journal of Research in Personality.

[B128-behavsci-16-01092] Russo F., Thaut M., Hodges D. (2019). Multisensory processing in music. The Oxford handbook of music and the brain.

[B129-behavsci-16-01092] Schiavio A., van der Schyff D. (2018). 4E music pedagogy and the principles of self-organization. Behavioral Sciences.

[B130-behavsci-16-01092] Schneck D., Berger D. (2010). The music effect. Music physiology and clinical applications.

[B131-behavsci-16-01092] Serafine M. (1988). Music as cognition. The development of thought in sound.

[B132-behavsci-16-01092] Sessions R. (1971). The musical experience of composer, performer, listener.

[B133-behavsci-16-01092] Shamma S. (2001). On the role of space and time in auditory processing. Trends in Cognitive Sciences.

[B134-behavsci-16-01092] Shapiro L. (2014). The Routledge handbook of embodied cognition.

[B135-behavsci-16-01092] Shepard R., Kubovy M., Pomerantz R. (1981). Psychophysical complementarity. Perceptual organization.

[B136-behavsci-16-01092] Shepard R. (1982a). Geometrical approximations to the structure of musical pitch. Psychological Review.

[B137-behavsci-16-01092] Shepard R., Deutsch D. (1982b). Structural representations of musical pitch. The psychology of music.

[B138-behavsci-16-01092] Shin Y., Proctor R., Capaldi E. (2010). A review of contemporary ideomotor theory. Psychological Bulletin.

[B139-behavsci-16-01092] Sievers E. (1924). Ziele und Wege der Schallanalyse. Zwei Vorträge von E. S., Heidelberg.

[B140-behavsci-16-01092] Silverman D. (2013). Sensorimotor enactivism and temporal experience. Adaptive Behavior.

[B141-behavsci-16-01092] Smalley D., Emmerson S. (1986). Spectro-morphology and Structuring Processes. The language of electroacoustic music.

[B142-behavsci-16-01092] Smalley D. (1997). Spectromorphology: Explaining sound-shapes. Organized Sound.

[B143-behavsci-16-01092] Smalley D., Gayou E. (2010). Spectromorphology in 2010. Denis Smalley: Polychrome portraits n. 15.

[B144-behavsci-16-01092] Smyth D. (2014). Infinity and givenness: Kant on the intuitive origin of spatial representation. Canadian Journal of Philosophy.

[B145-behavsci-16-01092] Stan M., McNulty M. (2022). Phoronomy: Space, construction, and mathematizing motion. Kant’s metaphysical foundations of natural science: A critical guide.

[B146-behavsci-16-01092] Stein B., Stanford T., Rowland B. (2014). Development of multisensory integration from the perspective of the individual neuron. Nature Reviews Neuroscience.

[B147-behavsci-16-01092] Stepp N., Turvey M. (2010). On strong anticipation. Cognitive Systems Research.

[B148-behavsci-16-01092] Sutherland D. (2014). Kant on the construction and composition of motion in the Phoronomy. Canadian Journal of Philosophy.

[B149-behavsci-16-01092] Thom R. (1972). Stabilité structurelle et Morphogenèse.

[B150-behavsci-16-01092] Thom R. (1989). Structural stability and morphogenesis: An outline of a general theory of models.

[B151-behavsci-16-01092] Thompson E. (2007). Mind in life: Biology, phenomenology, and the sciences of mind.

[B152-behavsci-16-01092] Todd N. (1992). The dynamics of dynamics: A model of musical expression. Journal of the Acoustical Society of America.

[B153-behavsci-16-01092] Todd P. (1999). Motion in music: A neurobiological perspective. Music Perception.

[B154-behavsci-16-01092] Truslit A. (1938). Gestaltung und Bewegung in der Musik. Ein tönendes Buch vom musikalischen Vortrag und seinem bewegungserlebten Gestalten und Hören.

[B155-behavsci-16-01092] Van der Meer W., Rao S. (2006). What you hear isn’t what you see: The representation and cognition of fast movements in Hindustani music. International Symposium Frontiers of Research on Speech and Music.

[B156-behavsci-16-01092] Varela F., Thompson E., Rosch E. (1991). The embodied mind: Cognitive science and human experience.

[B157-behavsci-16-01092] Vuillemin J. (1955). Physique et métaphysique kantiennes.

[B158-behavsci-16-01092] Walker C. (1995). Karl Jaspers and Edmund Husserl—III: Jaspers as a Kantian phenomenologist. Philosophy, Psychiatry, & Psychology.

[B159-behavsci-16-01092] Waszak F., Cardoso-Leite P., Hughes G. (2012). Action effect anticipation: Neurophysiological basis and functional consequences. Neuroscience & Biobehavioral Reviews.

[B160-behavsci-16-01092] Whitehead A. (1957). Process and reality. An Essay in cosmology.

[B161-behavsci-16-01092] Woelert P. (2007). Kant’s hands, spatial orientation, and the Copernican turn. Continental Philosophy, Review.

